# Fitness of Isogenic Colony Morphology Variants of *Pseudomonas aeruginosa* in Murine Airway Infection

**DOI:** 10.1371/journal.pone.0001685

**Published:** 2008-02-27

**Authors:** Elza Rakhimova, Antje Munder, Lutz Wiehlmann, Florian Bredenbruch, Burkhard Tümmler

**Affiliations:** 1 Clinical Research Group, OE6710, Hanover Medical School, Hanover, Germany; 2 Helmholtz Centre for Infection Research, Division of Cell Biology and Immunology, Braunschweig, Germany; University of Minnesota, United States of America

## Abstract

Chronic lung infections with *Pseudomonas aeruginosa* are associated with the diversification of the persisting clone into niche specialists and morphotypes, a phenomenon called ‘dissociative behaviour’. To explore the potential of *P. aeruginosa* to change its morphotype by single step loss-of–function mutagenesis, a signature-tagged mini-Tn*5* plasposon library of the cystic fibrosis airway isolate TBCF10839 was screened for colony morphology variants under nine different conditions *in vitro*. Transposon insertion into 1% of the genome changed colony morphology into eight discernable morphotypes. Half of the 55 targets encode features of primary or secondary metabolism whereby quinolone production was frequently affected. In the other half the transposon had inserted into genes of the functional categories transport, regulation or motility/chemotaxis. To mimic dissociative behaviour of isogenic strains in lungs, pools of 25 colony morphology variants were tested for competitive fitness in an acute murine airway infection model. Six of the 55 mutants either grew better or worse *in vivo* than *in vitro*, respectively. Metabolic proficiency of the colony morphology variant was a key determinant for survival in murine airways. The most common morphotype of self-destructive autolysis did unexpectedly not impair fitness. Transposon insertions into homologous genes of strain PAO1 did not reproduce the TBCF10839 mutant morphotypes for 16 of 19 examined loci pointing to an important role of the genetic background on colony morphology. Depending on the chosen *P. aeruginosa* strain, functional genome scans will explore other areas of the evolutionary landscape. Based on our discordant findings of mutant phenotypes in *P. aeruginosa* strains PAO1, PA14 and TBCF10839, we conclude that the current focus on few reference strains may miss modes of niche adaptation and dissociative behaviour that are relevant for the microevolution of complex traits in the wild.

## Introduction

*Pseudomonas aeruginosa* is a metabolically versatile γ-Proteobacterium, which inhabits terrestrial, aquatic, animal-, human-, and plant-host-associated environments [1]. This opportunistic pathogen causes chronic infections in the cystic fibrosis (CF) lung [2], [3] and has emerged as an important causative agent of nosocomial infections, particularly in ventilated patients in intensive-care units (ICU) [4]–[6].

*P. aeruginosa* isolates from acute and chronic airway infections of the human host display high phenotypic diversity [7], [8]. High frequency of phenotype switching is often the result of adaptive genetic diversification resulting in increased chances of bacterial survival in their niche [8]–[13]. Spatial compartmentalization in the supply of oxygen and nutrients and in the exposure to host inflammatory responses is associated with the diversification of *P. aeruginosa* into morphotypes and the establishment of niche specialists [7]–[9], [12]–[16]. Typical morphotypes in the CF lung are small colony variants (SCVs) [9], [17]–[19], alginate-overproducing mucoid variants [14], [15], [20]–[22], colourless variants [23] or colonies with visible autolysis [24] or autoaggregative behaviour [19], [24], [25].

Growth and morphology are easy-to-follow phenotypic traits of organismal adaptation that may or may not be genetically fixed by mutation and selection. The biological fitness of isogenic variants is not necessarily equal, but depends on the environment in which the organisms live [26]. Mucoid *P. aeruginosa* variants preferentially grow in biofilms under microaerophilic or anaerobic conditions [14], [15]. Clinical SCV isolates were described to display increased fitness in stationary phase, better biofilm-forming capabilities and high adherence to airway epithelial cells [9], [19]. Even autolysis, which might seem unambiguously detrimental to an unicellular organism, is an adaptive behaviour of *P. aeruginosa* mediated by overproduction of the quinolone PQS being an extracellular signal increasing the stringent response and the formation of protective biofilm by released DNA after the cells' lysis [9], [24], [27], [28]. Moreover, c-di-GMP levels regulate the differentiation of *P. aeruginosa* populations into macroscopic cell aggregates and planctonic cells [29].

Airway infections with *P. aeruginosa* are major determinants of morbidity and mortality for ventilated patients at ICU [4] and individuals with CF [2], [3], but the time scales of adaptation are different. *P. aeruginosa* rapidly diversifies within a few days in the airways of intubated patients in traits of virulence and antimicrobial resistance [5], [6]. In the CF lung, however, the colonizing *P. aeruginosa* clone will diversify in morphotype and lifestyle concurrently with airway remodelling and dedifferentiation [3] and sequentially accumulates mutations over a period of months to decades [8], [30], [31].

In this paper, we describe the genetic repertoire of *P. aeruginosa* to generate morphology variants by single loss-of-function mutations. By utilizing the approach of signature tagged mutagenesis (STM) [32]–[34], colony morphology variants were identified by screening a STM minitransposon library grown under different culture conditions *in vitro*. Pools of morphology variants were then tested in an acute murine airway infection model [35], [36] to evaluate their competitive fitness to grow *in vivo* and to breach epithelial barriers. An unexpectedly large number of genes was identified which can promote the adaptation to a mammalian niche via the modulation of the morphological phenotype by single transposon insertion.

## Results

### Selection of the *P. aeruginosa* strain

The STM mini-Tn*5* transposon library was generated in the *P. aeruginosa* strain TBCF10839. This strain was selected for various reasons. First, it belongs to a major clone in the *P. aeruginosa* population [37]. Second, it is part of a contagious cluster that had caused numerous outbreaks at ICUs and the ward for burn wounds at the authors' institution and had spread among patients of the local CF clinic by nosocomial acquisition [38]. TBCF10839 was isolated from an individual with CF at a point of time when he suffered from recurrent pulmonary exacerbations of his chronic *P. aeruginosa* infection. Transcriptome [39] and proteome analyses [40] indicated that TBCF10839 orchestrates many more metabolic and signalling pathways upon exposure to inanimate and animate stressors than the sequenced reference strain PAO1 [41]. Being a strong producer of virulence effector proteins and of siderophores, quinolones and phenazines, it is pathogenic for *Drosophila melanogaster* and *Caenorhabditis elegans* and causes substantial airway pathology in mice and rats after intratracheal instillation [34]. TBCF10839 is more virulent than the genetic reference strain PAO1 in infection models and can colonize naive murine airways [34]. The latter feature could not be established with strain PA14 [42] that depending on the dose was either cleared or lethal within a day (A. Bragonzi, personal communication). Hence, TBCF10839 that was derived from a chronic CF lung infection, was judged to be the more suitable reference strain for studies on morphotype variation and airway adaptation than the sequenced burn wound isolates PAO1 [41] and PA14 [42]. Being a mucoid strain, TBCF10839 had already acquired the morphological signature of chronic airway colonizers, and hence we expected that single hits in the genome should generate more informative morphological variants than the same approach in strains PAO1 or PA14.

### Construction and screening of the STM library

The STM library was constructed with the plasposon pTnModOGm [43] carrying variable V_40_ (V = A, G, C) signature tags (Text S1). The plasposon was randomly introduced into the TBCF10839 chromosome by triparental mating. Since no more than 5% of the cfu of each conjugation experiment were incorporated into the library, the redundancy of identical clones could be kept below 5%. The mutants were grown on M9 minimal medium with 0.5% glycerol (w/v) as sole carbon source in order to counterselect auxotrophic transposon mutants in housekeeping genes.

The 3,500 transposon mutants of the library were assayed for colony morphology variants. The mutants were plated to grow on LB agar at 4°C, 22–25°C, 37°C or 42°C or to grow at 37°C on blood agar or agar with minimal medium, iron-depleted LB, iron supplemented LB or with LB supplemented with the dye Congo red. Morphology was documented by shape, size, margin, colour and texture of the colonies. These growth conditions were chosen to mimic some key features of the lifestyle of the cosmopolitan *P. aeruginosa* in the wild [1]. The temperatures from 4°C to 42°C cover the full range of growth of the psychrotolerant organism. The minimal medium represents the nutrient-poor aquatic habitats where *P. aeruginosa* typically grows better than most microbial competitors [1]. Blood agar was taken as a surrogate for septicemia, the most devastating infection in the human host [4]. The nutrient-rich LB medium should approximate the animal mucosa including the chronically infected CF lung. The nutrients in the latter environment have been analyzed in depth for the proximal [12], but not the distal airways so that we considered the standard LB medium to be as informative as any more sophisticated sputum medium [44], [45]. The comparison of iron-depleted with iron-supplemented media was intended to explore the impact of the extremes in the availability of this essential micronutrient on morphotype [1]. Finally, Congo red was chosen to visualize differences in the exopolysaccharide matrix between wild type and mutants. Altogether, the different media provided information to what extent colony morphology was robust or dependent on growth conditions.

After three rounds of repeated screenings, there remained 55 mutants with a morphotype other than wild type (Table S1). When these 55 mutants were individually streaked on plates, a variable morphotype of individual colonies was noted for 25 mutants. The other 30 mutants exhibited robust morphotypes that were classified into eight categories (Table 1).

**Table 1:** *P. aeruginosa* TBCF10839 Tn*5* minitransposon colony morphology mutants.

Map position of Tn*5*: gene number in PAO1 genome	Type of morphology#	Gene name	Gene annotation
**Stable morphotypes distinct from wild type**			
PA0424	A	*mexR*	Multidrug resistance operon repressor MexR
PA2028	A	*-*	Probable transcriptional regulator
PA2122	A	*-*	Hypothetical protein
PA3462	A	*-*	Probable sensor/response regulator hybrid, transcriptional regulatory protein
PA3748	A	*-*	Conserved hypothetical protein; putative magnesium and cobalt transporter (CorB)
PA4190	A^+^	*pqsL*	Probable FAD-dependent monooxygenase
PA4489	A	*-*	Conserved hypothetical protein
PA5524	A	*-*	Probable short-chain dehydrogenase
-	A	*topA*	Topoisomerase 1A, *P. aeruginosa* 2192: genomic island PAGI-2, *P. aeruginosa* strain C.
-	A	*fpvA*	Siderophore receptor for type III ferripyoverdine, *P. aeruginosa* strain 59.20
-	A	*phiCTXp40*	Pseudomonas phage phiCTX, hypothetical protein, ORF37
PA0999*	B	*pqsD*	3-oxoacyl-[acyl-carrier-protein] synthase III
PA1003*	B	*mvfR*	Transcription regulator
PA2361	B	-	Hypothetical protein
PA4915	B	*-*	Probable chemotaxis transducer
PA2537	C	*-*	Probable acyltransferase
PA2579	C	*kynA*	Tryptophan 2,3-dioxygenase
PA2838	C	*-*	Probable transcriptional regulator
PA4734	C	*-*	Hypothetical protein
PA4552*	D	*pilW*	Type IV fimbrial biogenesis protein
PA4554*	D	*pilY1*	Type IV fimbrial biogenesis protein
PA0413	E	*chpA*	Chemotaxis protein
PA0415	E	*chpC*	Chemotaxis protein
PA1846	E	*cti*	Cis/trans isomerase
PA4954	E	*motC*	Membrane protein, part of the torque generator of the flagellar motor
PA2388	F	*fpvR*	Transcriptional regulator
PA2391	F	*opmQ*	Probable outer membrane protein precursor
PA3194*	G	*edd*	Phosphogluconate dehydratase
PA4640*	G	*mqoB*	Malate:quinone oxidoreductase
-	H	*-*	No homologies in PAO1 genome, GC low region at pos. 3291786–3291972 in a genomic island of the *P. aeruginosa* 2192 genome
**Unstable mutant morphotypes with rapid reversion to wild type morphotype**			
PA0482	-	*glcB*	Malate synthase G
PA0728	-	*-*	Probable bacteriophage integrase
PA0785*	-	*azoR*	Azobenzene reductase
PA0920	-	*-*	Hypothetical membrane protein
PA1589	-	*sucD*	Succinyl-CoA synthetase alpha chain
PA1633	-	*kdpA*	Potassium-transporting ATPase
PA1823	-	*-*	Hypothetical protein, predicted NADH pyrophosphatase
PA2706	-	*-*	Hypothetical protein
PA2946	-	*-*	Hypothetical protein; predicted integral membrane protein
PA3012	-	*-*	Hypothetical protein
PA3238	-	*-*	Hypothetical protein
PA3239	-	*-*	Conserved hypothetical protein, predicted surface lipoprotein (VacJ)
PA3804	-	*-*	Hypothetical protein
PA4131*	-	*-*	Probable iron-sulfur protein
PA4703	-	*-*	Hypothetical protein; predicted regulator of competence-specific genes (TfoX)
PA4797	-	*-*	Probable transposase.
PA4949	-	*-*	Conserved hypothetical protein; predicted sugar kinase
PA4951	-	*orn*	Transcription, RNA processing and degradation, oligoribonuclease
PA5121	-	*-*	Hypothetical membrane protein; predicted small-conductance mechanosensitive channel (MscS)
PA5231	-	*-*	Probable ATP-binding/permease fusion ABC transporter
PA5546	-	*-*	Conserved hypothetical protein; predicted cyclopropane fatty acid synthase (Cfa)
PA5563	-	*soj*	Chromosome partitioning protein
-	-	*-*	No homologies in PAO1 genome
-	-	*-*	Promoter region of PA1266 gene
-	-	*-*	Promoter region of PA3782 gene

# Morphotype: **A-**shiny autolysis, A^*^-shiny autolysis, but not white on iron supplemented medium; **B**–white colony on blood agar and iron supplemented media, on Congo red agar the colour is concentrated in the center of colony; **C**–light rose or colourless on Congo red agar, **D–**autolysis, but no shine, **E**-non mucoid at room temperature; **F–**orange pigment in the center of colony; **G–**small colony; **H**–highly mucoid.

* Mutant has been complemented in trans. Complementation of transposon insertion mutant with the recombinant plasmid carrying the respective full-length gene plus promoter reverted the mutant morphotype to colony morphology of the wild type TBCF10839 strain.

The insertion site of the plasposon was identified for all 55 mutants by sequencing of PCR products generated by the Y linker method [46] (Table 1). The plasposon had inactivated a PAO1-homologous sequence in 50 TBCF10839 mutants. The average diversity from the PAO1 sequence was just 0.2%. Four sequences were ascribed to phage phiCTX [47], two genomic islands known from *P. aeruginosa* strain 2192 (GI 84328724) and to a non-PAO type of the pyoverdine receptor FpvA [48], respectively. One sequence was not homologous to any *P. aeruginosa* sequence in the database. Of the 55 genes, properties of the encoded product have been characterized for 19 genes. A functional category could be ascribed *in silico* to 16 genes, whereas 20 ORFs encode hypothetical proteins of yet unknown function. The role of the gene products on colony morphology had so far only been investigated for PqsL, PqsD and MvfR that all are involved in the biosynthesis or regulation of 4-hydroxy-2-alkylquinolines (HAQs), the most prominent representative of which being the intercellular communication molecule 3,4-dihydroxy-2-heptylquinoline PQS [24], [49]–[55].

A subset of mutants was complemented with the full length gene *in trans.* These mutants were harbouring the transposon in a functionally characterized gene and were endowed with either lower or higher fitness of survival in murine lungs (see below). Eight mutants (Table 1) fulfilled these criteria and in all eight cases the mutant morphotype was reverted to the wild-type phenotype of strain TBCF10839 by complementation *in trans*.

### Colony morphology variants of P. aeruginosa

**Figure 1:**
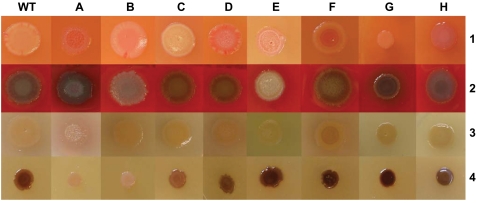
Types of colony morphology of *P. aeruginosa* TBCF10839 mini-Tn*5* transposon mutants on LB agar with Congo red (1), blood agar (2), LB agar (3), LB agar supplemented with 4 mM FeSO_4_ (4): WT, TBCF10839 strain; A, shiny autolysis; B, white colony on blood agar and iron supplemented media, on Congo red agar the colour is concentrated in the center of colony; C, light rose or colourless on Congo red agar; D, soft autolysis, no shine; E, non mucoid structure at room temperature; F, orange pigment in the center of colony; G, small colony size; H, highly mucoid. Transposon insertions into the TBCF10839 homolog of PA2028 (A), PA1003 (B), PA2579 (C), PA4554 (D), PA0415 (E), PA2388 (F), PA4640 (G), GC low region at pos. 3291786–3291972 in a genomic island of the *P. aeruginosa* 2192 genome (H).

Representative phenotypes of the eight robust morphotypes are shown in Figure 1. Glossy colonies with an inner circle of lysed cells (Figure 1A) represented the most frequent morphotype in our panel (type A, Table 1). The autolysis was visible after 24 h of incubation at 37°C on all tested media. In case of PqsL which is a negative regulator of HAQ biosynthesis [24], the autolysis of the *pqsL^−^* mutant could be attributed to the uncontrolled overproduction of bactericidal HAQs, but the etiology of autolysis in the other mutants remained elusive. Colonies of wild-type strains are typically brownish in high-iron medium (4 mM FeSO_4_), but all type A colony morphology mutants other than PqsL were colourless indicating that iron uptake was compromised. The fact that the pyoverdine receptor FpvA, the major uptake system for iron in *P. aeruginosa* [48], [56], is amongst these mutants, is consistent with this interpretation.

**Figure 2:**
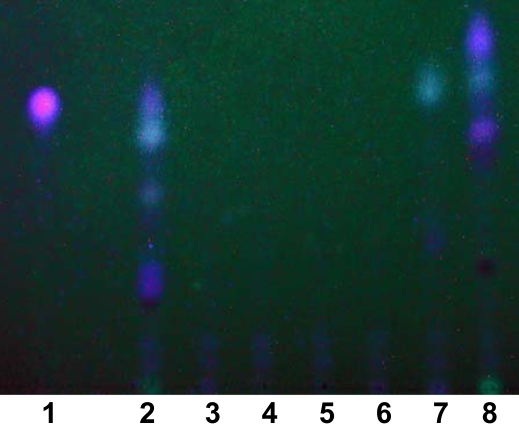
Thin layer chromatogram of HHQ metabolites. Extracts isolated from TBCF10839 (lane 2) and its isogenic transposon mutants Tn*5*::PA0999 (lane 3), Tn*5*::PA1003 (lane 4), Tn*5*::PA2361 (lane 5), Tn*5*::PA4915 (lane 6), Tn*5*::PA2838 (lane 7), Tn*5*::PA4190 (lane 8), synthesized PQS (standard, lane 1).

Type B colony morphology mutants (Figure 1B) appeared white on iron supplemented medium and blood agar, were impaired in hemolytic activity and accumulated the dye Congo red in the center of the colony. Congo red is known to bind extracellular matrix components and is taken as a surrogate marker for biofilm formation [57], [58]. All type B mutants secreted proteases (data not shown) [59], but lacked HAQ production [49] (Figure 2). The pleiotropic B phenotype was not only caused by the inactivation of members of the HAQ biosynthesis operon (*pqsD* and *mvfR*) [24], [49], [51], [53], [54], but also by that of two yet uncharacterized genes (PA2361, PA4915) (Table 1). PA2361 and PA4915 harbour sequence signatures of metalloproteinases and chemotaxis proteins and have orthologs in almost all sequenced Pseudomonas genomes and numerous other gamma-proteobacteria with an overall sequence similarity of 30–40%. Our data indicate that PA2361 and PA4915 gene products are essential for the production of quinolones which adds a further layer of complexity on the regulation of HAQ production.

Colony type C mutants were light rose or colourless on Congo red agar (Figure 1C) suggesting that biofilm formation was affected. The phenotype was shared by mutants of metabolic genes (PA2537, PA2579), a LysR transcriptional regulator (PA2838) and a conserved hypothetical (PA4734) that has numerous orthologs in ‘honorary pseudomonads’, namely the metabolically related Burkholderia, Ralstonia and Xanthomonas bacteria.

Mutants of the non-piliated TB strain in the pilin biosynthesis genes *pilY1* and *pilW* showed colonies with soft autolysis on LB agar, but not on blood agar or on plates supplemented with or depleted of iron (Figure 1D). Autolysis became visible by 9 hours of incubation and was maximal by 48 hours. This yet undescribed medium-dependent phenotype of cell lysis points to pili-unrelated roles of PilY1 and PilW for the *P. aeruginosa* cell that deserves further investigation.

In four mutants the alginate-overproducing TB strain had reverted to a non-mucoid phenotype (Figure 1E) and in one mutant transposon mutagenesis in a region that is homologous to a 38 kb genomic island of *P. aeruginosa* 2192, induced an even stronger mucoid morphotype (Figure 1H). Two mutants turned orange on Congo red agar due to the knock-out of genes (*fpvR, opmQ*) of the pyoverdine locus [48], [56] (Figure 1F). Furthermore, colonies were smaller when transposon mutagenesis had hit two core genes of energy and carbohydrate metabolism, the phosphogluconate dehydrogenase Edd that converts 6-P-gluconate to 2-keto-3-deoxy-6-P-gluconate in the Entner-Douderoff pathway [60], and the malate:quinone oxidoreductase MqoB that catalyzes the conversion of malate to oxaloacetate in the citric acid/glyoxylate cycles [61] (Figure 1G).

### Survival of colony morphology variants in a murine airway infection model

**Figure 3:**
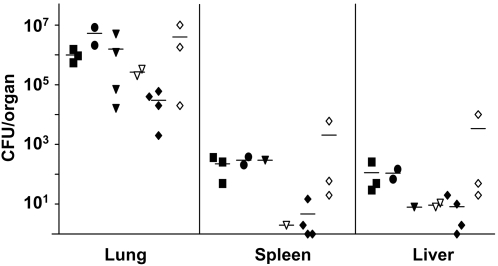
Total CFU of *P. aeruginosa* TBCF10839 STM colony morphology mutants recovered from murine organs 48 h after intratracheal instillation of up to 25 differentially tagged mutants. Each data point represents the recovery of bacteria from one organ of one animal. Different test sets of TBCF10839 STM mutants are differentiated by symbol. The in total 55 different mutants were first tested in different combinations in a series of four separate experiments (closed square, closed circle, closed triangle, open triangle) whereby each mutant per test set haboured a different oligonucleotide tag. After the abundance of individual mutants had been semiquantitatively evaluated by dot-blot hybridizations (see Figure 4), the mutants with the lowest (closed diamonds, 10 mutants) and highest survival (open diamonds, 10 mutants), respectively, were pooled and tested again.

**Figure 4:**
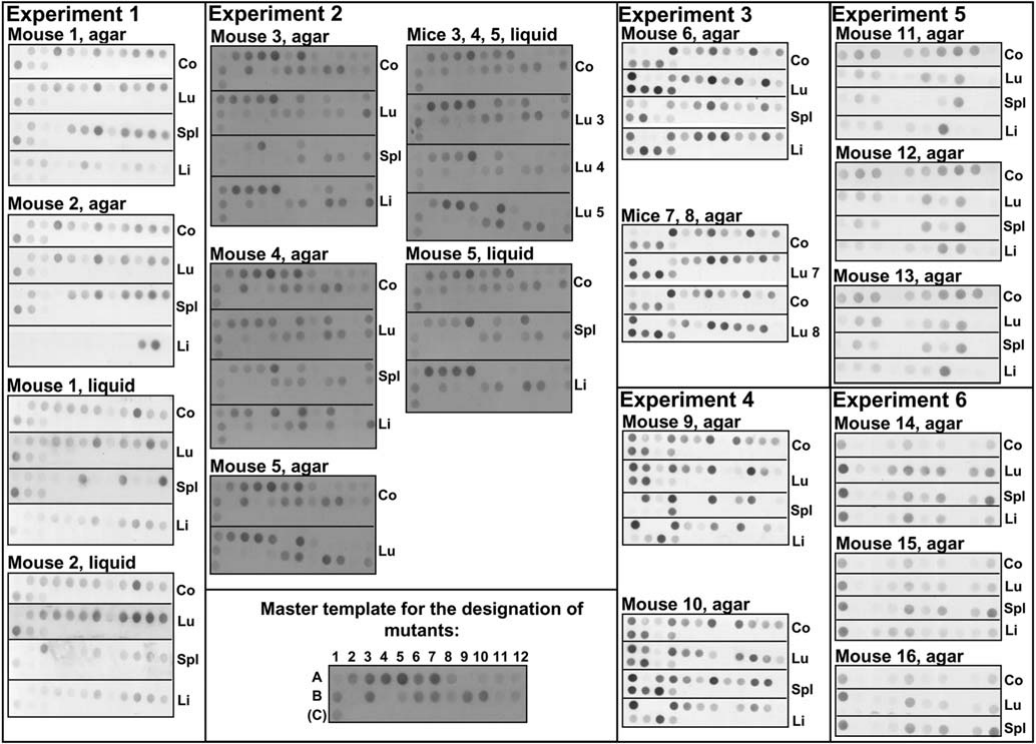
Dot blot hybridization of oligonucleotide signature tags to determine the survival of individual *P. aeruginosa* TBCF10839 colony morphology mutants in competition experiments. DIG-labeled, HindIII-digested signal sequences isolated from bacteria not subjected to selection (control, co) or from bacteria recovered from organs after murine infection experiments (lung, lu; spleen, spl; and liver, li) were hybridized onto dot blots of the signal sequences of the pTnModOGm SigTag. The recovered bacteria had been either cultured on solid LB agar (designated ‘agar’ in the Figure) or in LB broth (designated ‘liquid’ in the Figure). Experiments are numbered as in Figure 3. First, the total pool of colony morphology variants was tested in different combinations in experiments 1, 2, 3 and 4. Ten mutants each with the lowest and highest survival were then retested in experiments 5 and 6, respectively. The PA number of the transposon-inactivated gene that carries the signature tag complementary to the oligonucleotide on the dot blot is given in Table S2. Mutants were arranged as indicated in the master template.

To mimic the dissociative behaviour of *P. aeruginosa* in lung infections, the 55 isogenic colony morphology variants were tested for their competitive fitness to survive in murine airways and to spread to other organs. Pools of 25 mutants each of which harbouring different signature tags were instilled into murine airways and bacteria were recovered 48 hours later from lungs, spleen and liver. After four experiments the mutants with the least and the highest survival were separately tested in a fifth and sixth round (Figure 3). Figure 4 shows the outcome of the infection experiments documented by the dot blot hybridization signals of the signature tags of all tested transposon insertion mutants. Table 2 lists the 15 colony morphology mutants that grew better (six mutants, category 1) or worse (six mutants, category 2) than their competitors *in vivo* or that were globally compromised in growth (three mutants, category 3).

**Table 2:** STM competition experiments. *P. aeruginosa* TBCF10839 colony morphology mutants with higher or lower fitness in acute murine airway infection.

Map position of Tn*5* mutant: gene number in PAO1	Annotation
**Category 1: gain of function (enhanced survival ** ***in vivo*** **)**	
PA4131	Probable iron-sulfur protein
PA4552	*pilW* , type 4 fimbrial biogenesis protein
PA4554	*pilY1*, type 4 fimbrial biogenesis protein
PA4734	Conserved hypothetical protein
PA4954	*motC*, chemotaxis protein
PA5546	Hypothetical protein; predicted cyclopropane fatty acid synthase (Cfa)
**Category 2: loss of function (reduced survival ** ***in vivo*** **)**	
PA0999	*pqsD*, 3-oxoacyl-[acyl-carrier-protein] synthase III
PA2537	Probable acyltransferase
PA2706	Hypothetical protein
PA2838	Probable transcriptional regulator
PA3239	Hypothetical protein; predicted surface lipoprotein (VacJ)
PA4640	*mqoB,* malate:quinone oxidoreductase
**Category 3: non-competitive (no survival ** ***in vitro*** ** and ** ***in vivo*** **)**	
PA0785	*azoR*, azobenzene reductase
PA3194	*edd*, phosphogluconate dehydratase
phiCTX	*Pseudomonas* phage phiCTX, hypothetical protein

Ten of the 15 mutants belonged to the group with robust morphotypes which included all type D and G, three type C, one type B, one type E and one mutant of the most abundant category A of strains with glossy autolysis. In the latter singular case transposon mutagenesis had hit the phiCTX phage [47] that probably made this strain vulnerable to the attack of phage –proficient competitors so that it could not survive. Besides this phage mutant, the susceptibility to autolysis apparently conferred neither an advantage nor disadvantage to the strains to persist in murine airways and to spread to other organs.

High fitness *in vivo* was associated with a change of extracellular texture (PA4734, *motC*), impaired swarming and swimming (*motC*) or predisposition to cell lysis (*pilW, pilY1*). The wild-type TB strain is non-piliated due to a deletion in the *pilQ* gene [62] and hence any secondary mutations in pilin biogenesis genes would be phenotypically silent with respect to pilin production. The autolysis of the mutants, however, points to further features of PilW and PilY1 that are unrelated to pilin biogenesis and should confer the higher fitness of the mutants in the STM infection experiments. Low fitness in murine airways was observed in metabolic- (PA2537, PA2838; *mqoB*) or HAQ-deficient (*pqsD*) mutant morphotypes. In case of the globally compromised mutants, the transposon had disrupted a gene that is essential for survival in a community of isogenic TB strains. Not surprisingly mutants were carrying the transposon in metabolic genes. Although the mutants were not auxotrophic and could grow in pure cultures, they could not successfully compete for nutrients in a microbial community of isogenic mutants.

### Infection experiments on isolated mutants

To differentiate between the virulence for the host and the fitness within the bacterial community, representative mutants of the three categories were retested separately in infection experiments. To simplify the interpretation of the data, we selected mutants in which the transposon had targeted genes whose encoded function had already been studied in *P. aeruginosa*.

**Figure 5:**
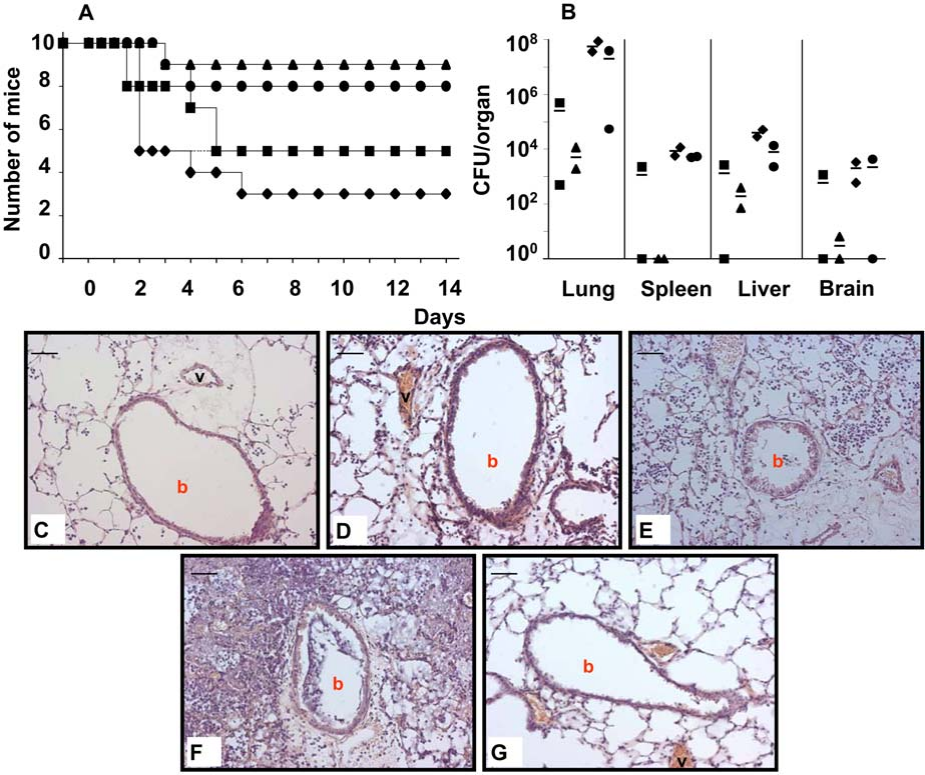
(A) Survival of mice whose airways had been inoculated at day 0 with 7.5×10^6^ CFU of *P. aeruginosa* TBCF10839 (closed square) or of the isogenic transposon mutants *P. aeruginosa* TBCF10839 Tn*5*::PA4640(*mqoB*) (closed circle), *P. aeruginosa* TBCF10839 Tn*5*::PA3194(*edd*) (closed triangle) or *P. aeruginosa* TBCF10839 Tn*5*::PA4954(*motC*) (closed diamond). (B) CFU recovered from murine organs 48 h after intracheal instillation of TBCF10839 (closed square), Tn*5*::PA4640 (closed circle), Tn*5*::PA3194 (closed triangle) or Tn*5*::PA4954 (closed diamond). (C, D, E, F, G) Lung histology 48 h after infection with Tn*5*::PA4640 (C), Tn*5*::PA3194 (D), Tn*5*::PA4954 (E), TBCF10839 wild type (F) or vehicle control (30 µL PBS) (G). Hematoxylin-eosin stain; original magnification×200, bar 50 µm. b, bronchus; v, vessel. The photomicrographs show normal tissue with a few alveolar macrophages and neutrophils (C), minimally intense peribronchiolar and perivascular inflammation (D), moderate inflammation (alveoli filled with inflammatory cells, but only few inflammatory cells within bronchi) (E), profound inflammation (infiltration of bronchi with leukocytes, destructed lung tissue) (F) and normal lung parenchyma (G).

Of the tested strains, the category I *motC^−^* mutant was most virulent and most heavily growing in murine organs (Figure 5). Its high survival *in vivo* could be attributed to its competence to colonize murine lungs and to breach the epithelial barrier. The *P. aeruginosa* genome contains dual sets of *motAB*-like genes [63]. The encoded membrane proteins that use membrane potential to conduct ions are required for the rotation of the flagellar motor [63]. Disruption of *motC* caused phenotypes that prima facie are unrelated to the established role of MotAB-like proteins in swimming and swarming, i.e. a switch in morphotype and better growth and survival in the mammalian host. These latter phenotypes, however, are consistent with the observation that a *motC* mutant of the *P. aeruginosa* CF isolate 4020 was resistant to phagocytosis by murine macrophages which represent the primary defense against *P. aeruginosa* in airways [64]. Maintenance of virulence, however, was not generally observed in category I morphotype mutants. Infection with the *pilY1^−^* mutant induced a strong acute inflammatory response in murine airways, but no mortality (data not shown).

The examined category II (*mqoB*) [61] and III mutants (*edd*) [60] were attenuated in virulence and caused less inflammation in murine lungs than wild type TBCF10839 (Figure 5). The *mqoB^−^* mutant was recovered in wild type frequencies from lungs, liver, spleen and brain, whereas the *edd^−^* mutant was more efficiently eliminated than TBCF10839 (Figure 5). Hence, the latter mutant was compromised in both fitness and virulence, and the former was attenuated in virulence and its competitiveness in the microbial community *in vivo*, but proficient to grow in the community *in vitro* and to survive as solitary organism *in vivo*.

### Metabolic fitness of isolated mutants

**Figure 6:**
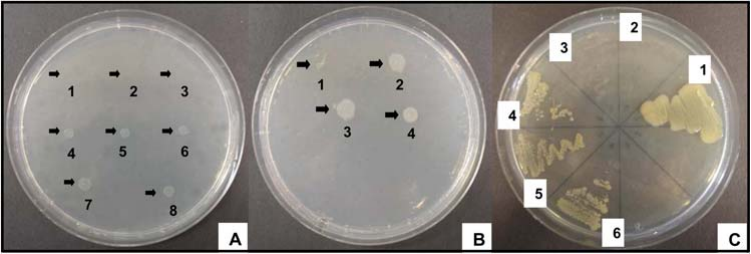
(A) Growth on minimal medium with 25 mM ethanol after 24h of incubation of *P. aeruginosa* TBCF10839 transposon insertion mutant Tn*5*::TB*mqoB* (1), *P. aeruginosa* PA14 MrT*7*::PA14*mqoB* (2), *P. aeruginosa* TBCF10839 Tn*5*::TB*mqoB* transformed with pUCP20 plasmid (vector control) (3), *P. aeruginosa* TBCF10839 Tn*5*::TB*mqoB* mutant complemented with pUCP20::TB*mqoB* (carrying the HindIII/SacI PCR product bearing the *mqoB* gene) (4, 5, 6), TBCF10839 (7) and PA14 (8) wild type strains. (B) Growth on minimal medium with 25 mM glucose after 24h of incubation of transposon insertion mutants Tn*5*::TB*edd* of strain TBCF10839 (1), MrT*7*::PA14*edd* of strain PA14 (2), TBCF10839 (3) and PA14 (4) wild type strains. (C) Growth on minimal medium with 25 mM glucose after 48h of incubation of *P. aeruginosa* strain TBCF10839 (1) and the isogenic transposon insertion mutants Tn*5*::TB*edd* (2), Tn*5*::TB*edd* transformed with pUCP20 plasmid (vector control) (3), Tn*5*::TB*edd* mutant complemented with pUCP20::TB*edd* carrying the HindIII/SacI PCR product bearing the *edd* gene (4, 5, 6).

**Figure 7:**
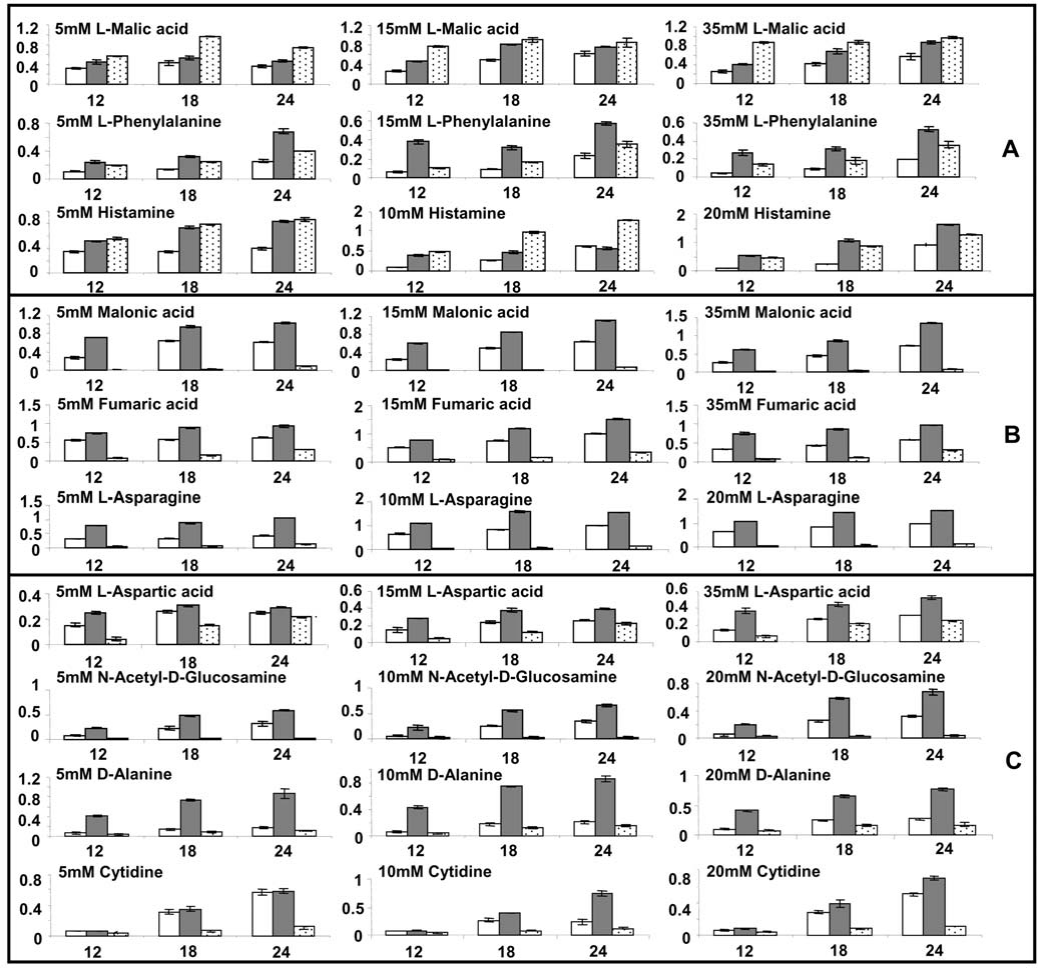
Growth of *P. aeruginosa* strains TBCF10839 (open bar), PAO1 (grey bar) and TBCF10839 Tn*5*::TB*motC* (A, dashed bar), TBCF10839 Tn*5*::TB*mqoB* (B, dashed bar), TBCF10839 Tn*5*::TB*edd* (C, dashed bar) at 37°C in standard minimal mineral medium supplemented with the indicated nutrients as carbon or nitrogen source. The ordinate indicates the optical density at 490 nm. The number below the triple bars indicates the time of culturing of 12 h (left), 18 h (middle) and 24 h (right). The initial bacterial inoculum had an OD_578_ of 0.02 in 100 µL minimal medium supplemented with the indicated carbon or nitrogen source. All growth experiments were performed in triplicate in 96-well plates.

One major factor that could account for the differential fitness of the three tested category I, II and III mutants in murine lungs could originate from their differential metabolic capacity. In accordance with the literature [60], [61] the *mqoB^−^* mutant was not capable to utilize ethanol and the *edd^−^* mutant did not grow on glucose as sole carbon source (Figure 6). To gain a more thorough insight into the influence of the transposon insertions on nutrient utilization, a metabolic profiling of *P. aeruginosa* PAO1, TBCF10839 and its isogenic *motC^−^, edd^−^* and *mqoB^−^* mutants was performed with commercial BiOLOG phenotype microarrays (Tables S3, S4, S5). If the screening provided evidence for the different utilization of a carbon, nitrogen, phosphorus or sulfur source, growth experiments were repeated in-house under carefully controlled conditions (Figure 7, Figure S1).

First, we noted a faster turnover of nutrients with the TBCF10839 *motC^−^* mutant. The mutant more efficiently metabolized malic acid as a carbon source and ammonia, histamine or phenylalanine as a nitrogen source than the wild type strain (Figure 7A). Particularly the latter two compounds should be present in ample amounts in a mammalian habitat such as the lungs. Thus, the *motC^−^* mutant showed a gain of metabolic fitness to inhabit the atypical niche of the mammalian airways consistent with its gain-of-fitness phenotype in the STM competition experiments.

The *mqoB^−^* mutant lacks the most potent enzyme for the conversion of malate to oxaloacetate. Correspondingly, the mutant was severely compromised in the utilization of malonate, fumarate and acetate as C-source and of the derivative of a C-4 compound asparagine as N-source (Figure 7B). The inactivation of a key enzyme of the citric acid and glyoxylate cycles make it plausible why the transposon insertion in *mqoB* reduced bacterial fitness to persist and grow *in vivo*.

Edd is a key enzyme of the Entner-Douderoff pathway. Consistent with the enzymatic activity of Edd, the mutant was not capable to metabolize the sugar derivative N-acetyl-glucosamine as a nitrogen source (Figure 7C). In addition, the utilization of aspartate, asparagine, alanine and cytidine was impaired demonstrating that the inactivation of *edd* by transposon mutagenesis had broad implications for the cellular metabolism. This global role of Edd fits with the observation that the fitness of the *edd* mutant was compromised *in vitro* and *in vivo*.

## Discussion

Chronic airway infections with *P. aeruginosa* are frequent in patients with CF [2], [3], bronchiectasis [65] or late stages of chronic obstructive pulmonary disease COPD [66]. Plating of respiratory tract specimens recovered from these patients will typically reveal numerous morphotypes, a phenomenon termed ‘dissociative behaviour’ by microbiologists more than forty years ago [7]. Already in this early pioneering paper drastic changes in colony morphology were noted during subculturing of the primary isolates indicating that the morphotype of *P. aeruginosa* is unstable and determined by many environmental cues. Research in this area has recently experienced a strong revival when the emergence of SCVs, autolysis and mucoidy could be associated with habitat-specific conditions of CF lungs [8], [9], [12], [14], [15], [17], [19], [20], [67], [68].

Colony morphology is an easy-to-follow phenotypic trait and hence the morphotype is used as a major criterion in the clinical microbiology laboratory to select isolates for further analysis. The underlying biology, however, is complex. The colony is a microbial community of genetically identical organisms whereby the individual cell faces a continuously changing microenvironment determined by the mass flow of nutrients and metabolites and the signals of its neighbours. Consequently, features of growth and aging, motility, secretion, extracellular matrix composition and cell-to-cell communication will govern the macroscopic appearance of a colony [69].

The dissociative behaviour of *P. aeruginosa* may result from the division of labour within the community or from the emergence of niche specialists or of selfish ‘cheaters’ [70]–[82], just to quote some obvious traits of adaptation. We were interested to know if and if yes, to what extent a single mutation could change both morphotype and biological fitness of *P. aeruginosa* in the atypical niche of mammalian lungs. A metabolically versatile and pathogenic isolate from chronically infected CF lungs [38] was chosen as the model organism because we hypothesized that such a strain had demonstrated its capability to persist in human airways and thus the perturbation by single hit mutagenesis should unravel the possible next steps of dissociative behaviour during niche adaptation.

Screening of a library of non-auxotrophic transposon mutants under different culture conditions *in vitro* uncovered that a knock-out in about 1% of the 5,500 ORFs of the *P. aeruginosa* genome led to a change of morphotype half of which was robust. In the other half environmental cues could rapidly modulate colony morphology in accordance with the complex etiology and rapid turnover of this phenotypic trait in nature [7], [8].

The colony morphology variants were allocated to eight distinguishable morphotypes all of which are regularly recovered from chronically infected CF lungs. Mucoidy [8], [14], [20], autolysis [24] and SCVs [9], [17], [19] are the most prominent variations but also colorless variants [23] and colonies with more subtle changes in the extracellular matrix that can be visualized with Congo red are typically seen among the primary isolates from respiratory specimens.

Half of the identified targets encode features of primary or secondary metabolism, and the other half is shared in equal portions by genes assigned to the categories of transport, regulation or motility/chemotaxis (Tables 1 & 2). Interestingly neither a known virulence effector protein nor a gene product with an established role for mucoidy or SCVs were detected in the screen. The TBCF10839 strain had acquired a robust mucoid morphotype in the CF lungs, and transposon insertion had led to a nonmucoid strain in four mutants, but none of the targets has reported previously to be associated with alginate biosynthesis or its regulation [1], [20]. Two targets belong to the machinery of chemotaxis and flagellar motility [1], [63]. Since the mutants switched back to the mucoid phenotype of the wild type strain by complementation *in trans*, we must assume a complex regulation of motility and alginate production in this strain that was not observed in the PAO1 reference strains (Table 3).

**Table 3:** Comparison of colony morphology of TBCF10839 and PAO1 transposon mutants that are inserted into homologous genes #.

PAO1 gene number of transposon insertion	Morphotype*	
	TBCF10839 transposon mutants	PAO1 transposon mutants**
PA2122+	A	not A; wild type PAO1
PA3462++	A	not A; wild type PAO1
PA3748++	A	not A; wild type PAO1
PA4190++	A	A
PA4489++	A	not A; wild type PAO1
PA0999++	B	B
PA1003++	B	B
PA2361++	B	not B; wild type PAO1
PA4915++	B	not B; wild type PAO1
PA4734++	C	not C; wild type PAO1
PA4552++	D	not D; wild type PAO1
PA4554++	D	not D; wild type PAO1
PA0413++	E	not E; wild type PAO1
PA0415++	E	not E; wild type PAO1
PA1846++	E	not E; wild type PAO1
PA4954++	E	not E; wild type PAO1
PA2388+	F	not F; wild type PAO1
PA2391++	F	not F; wild type PAO1
PA4640+	G	not G; wild type PAO1

# TBCF10839 homologues are 99.8% or more identical in sequence with the respective PAO1 genes.

* Morphotype: **A,** shiny autolysis; **B,** white colony on blood agar and iron supplemented media, on Congo red agar the colour is concentrated in the center of colony; **C,** light rose or colourless on Congo red agar; **D,** autolysis, but no shine; **E,** non mucoid structure at room temperature; **F,** orange pigment in the center of colony; **G,** small colony size.

** PAO1 mutants were provided by the University of Washington Genome Center “*Pseudomonas aeruginosa* PAO1 mutant collection” (http://www.genome.washington.edu) [84].

+ One PAO1 transposon mutant;

++ Two PAO1 transposon mutants with an insert at the 5′ end (mutant 1) or at the 3′ end (mutant 2) of the gene.

Autolysis [24], [27], [28], [67] divided into three subtypes was the most prominent morphotype in the collection of single mutants. Cell lysis is supposed to be a disadvantageous phenotype, however, all phage-competent autolytic mutants were not impaired in their fitness to grow in murine airways. For all other morphotypes, however, the association between colony morphology *in vitro* and survival in *vivo* was rather loose indicating that gene inactivation may modulate fitness by various modes, the change of morphotype being the most visible but not necessarily the most relevant feature *in vivo*. Moreover, knock-outs in unrelated pathways or regulons may converge to the same morphotype. The type B colony morphology mutants are an illustrating example. All mutants did not produce quinolones and phenazines *in vitro*. *PqsD* is a structural [24], [49] and *mvfR* the regulatory gene [50]–[53] of the HAQ biosynthesis operon [49], [54], but the targets of the other two type B mutants are not directly involved in quinolone or phenazine synthesis. They are probably members of a network that regulates the production and transport of these major secondary metabolites of *P. aeruginosa* some of which exhibit antimicrobial activity, modulate the host defense or act as bacterial signalling molecules [1]. Hence, the knock-out of an essential gene for biosynthesis should be detrimental for fitness, but the knock-out of a regulatory gene may be compensated by other regulons of the *P. aeruginosa* signalling network. Consistent with this interpretation, the PqsD knock-out was outcompeted by isogenic strains that were capable to synthesize quinolones, whereas the knock-out in the master regulatory gene *mvfR* was not compromised in its fitness *in vivo*. In summary, autolysis was an evolutionarily neutral trait in murine lungs, but defects in secondary metabolism could impede survival *in vivo*.

For us, the metabolic control of morphotype was the main and most unexpected result of the study. Colony morphology variants were caused most frequently by the inactivation of metabolic genes. The true proportion of metabolic gene knock-outs in colony morphology variants is probably even higher because a library was screened that had been counterselected for auxotrophic mutants.

**Figure 8:**
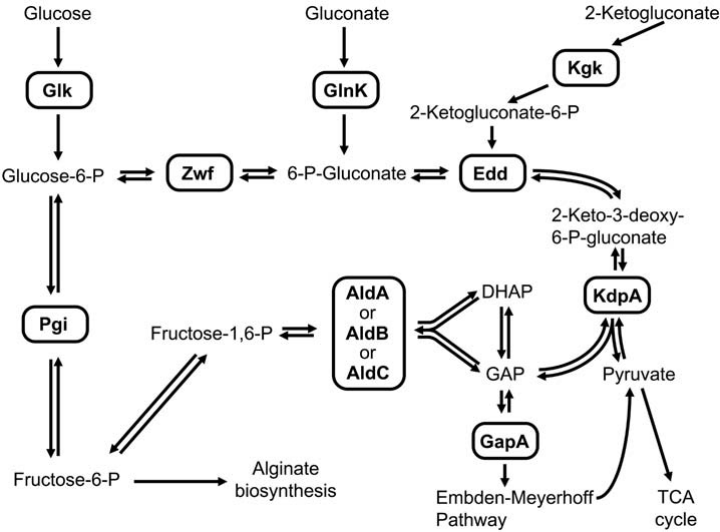
Central carbohydrate pathways in *P. aeruginosa*. An Edd knock-out is typically not able to grow on glucose as sole carbon source underpinning the dominant role of the Entner Douderoff pathway for sugar catabolism in *P. aeruginosa*. Carbohydrates are on the other hand no major preferred nutrients for *P. aeruginosa* [1] and knock outs in the central carbohydrate pathways do not appear as auxotrophic organisms, albeit they are compromised in their metabolic capacity and grow more slowly than the wild type. Consistent with the subordinate role of sugars as carbon source, the genes of sugar dissimilation are only weakly or moderately expressed (source: transcriptome data of strains PAO1, LES400, LES431 and TBCF10839 [39], [96]). Enzyme abbreviations: Ald, aldolase; Edd, 6-phosphogluconate dehydratase; GapA, glyceraldehyde-3-phosphate dehydrogenase; Glk, glucokinase; GlnK, gluconokinase; KdpA, 2-keto-3-deoxy-6-phosphogluconate aldolase; Kgk, 2-ketogluconate kinase; Pgi, phosphoglucoisomerase; Zwf, glucose 6-phosphate dehydrogenase.

**Figure 9:**
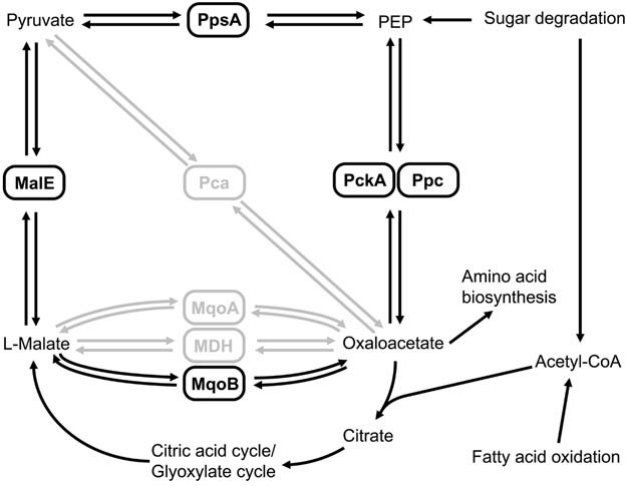
The malate–oxalacetate pathway. The malate:quinone oxidoreductase MqoB is the major enzyme for the conversion of malate to oxaloacetate. A knock-out of MqoB impairs the biosynthesis of sugars and amino acids. *P. aeruginosa mqoB^−^* mutants cannot grow on C2- (ethanol, acetate) or C4-compounds (malate, fumarate) as sole carbon sources indicating that the alternative pathways do not compensate the loss of MqoB. Faint fonts designate genes that were not expressed by strains PAO1 and TBCF10839 during growth in LB medium [39]. Enzyme abbreviations: MalE (PA3471), malic enzyme; MDH (PA1252), L-malate dehydrogenase; MqoA (PA3452), malate:quinone oxidoreductase; MqoB (PA4640), malate:quinone oxidoreductase; Pca (PA1400), pyruvate carboxylase; PckA (PA5192), phosphoenolpyruvate carboxykinase; Ppc (PA3687), phosphoenolpyruvate carboxylase; PpsA (PA1779), phophoenolpyruvate synthetase.

To understand of how single knock-outs of metabolic pathways translate into changes of morphotype and biological fitness, we selected colony morphology variants for metabolic phenotyping that were defective in two key enzymes of the citric acid cycle and the Entner-Douderoff pathway, MqoB and Edd. MqoB and Edd loss-of-function mutants could not utilize ethanol [61] or glucose as sole carbon source, respectively (Figure 6, Tables S4, S5). Edd (6-phosphogluconate dehydratase, EC 4.2.1.12) is involved in the carbon flow from glucose and gluconate into pyruvate and alginate [83] (Figure 8) which reasonably explains why an *edd^−^* mutant is less mucoid and out-competed *in vitro* and *in vivo*. The scenario is more complex for MqoB (Figure 9). The *P. aeruginosa* genome encodes one poorly expressed cytosolic NAD-dependent malate dehydrogenase (EC 1.1.1.37; PA1252) and two membrane-bound malate-quinone oxidoreductases (MqoA, MqoB). The two latter FAD-dependent enzymes catalyze the conversion of malate to oxaloacetate whereby the electrons are donated to quinones of the electron transfer chain. Under standard growth conditions *in vitro*, MqoB is more strongly expressed than MqoA both at the transcriptional (own unpublished data) and translational levels [61]. MqoB is the key enzyme for the production of oxaloacetate which is a precursor for the synthesis of amino acids and citrate. Malic enzyme and pyruvate carboxylase may bypass the lack of MqoB but this pathway of oxaloacetate synthesis is insufficient for growth if C2-compounds like acetate or ethanol are provided as sole carbon sources [61] (Figure 6). The MqoB mutant grew normally on LB within the microbial community of isogenic strains, but it grew slower on plates, was less virulent than wild type and was outcompeted in murine lungs. This data suggests that the proficient production of oxaloacetate is necessary for airway colonizing capacity and virulence of *P. aeruginosa*. In other words, biological fitness of *P. aeruginosa* requires that the turnover of acetyl-CoA, the key intermediate of sugar and fatty acid degradation, is not restricted by the insufficient supply of oxaloacetate.

When we retested the phenotype of transposon mutants found in TBCF10839 in publicly available transposon mutant collections of sequenced strains PAO1 [84] or PA14 [85], we noted that biochemical or physiological phenotypes of low complexity such as the presence of an enzyme or metabolite were similar in most, but not all tested cases (see examples in Figure 6). In contrast, the complex trait of a colony morphology variant found in a TBCF10839 transposon mutant was not reproduced by the insertion of a transposon into the homologous gene of the PAO1 strain in 16 of 19 examined loci indicating that the genetic background is essential for shaping complex phenotypes (Table 3). Similarly, although interclonal sequence diversity in *P. aeruginosa* is on the average just 0.5% [8], [86], subtle genetic differences may strongly influence the phenotype of complex traits. This observation is in accordance with the substantial variation of the transcriptome and proteome among genetically highly related strains with less than 0.01% sequence diversity among themselves [25], [39], [40], [87] and the inconsistent phenotype of pathogenicity genes [42]. When homologs of pathogenicity factors discovered in the genome island PAPI-1 of PA14 were retested in other strains, no correlation between the absence or presence of these genes with virulence was noted in worm infection models indicating that virulence is combinatorial in *P. aeruginosa* [42]. Our data shows that the same conclusion applies to the complex phenotype of colony morphology.

In summary, this and other studies demonstrate the important role of the genetic background on genotype-phenotype associations, at least for complex traits. Depending on the chosen *P. aeruginosa* strain, each functional genome scan by single gene disruptions will thus explore other areas of the evolutionary landscape. For example, the reversion of a mucoid to a non-mucoid phenotype that affected the fitness of the TBCF10839 strain (Table 2), will not be accessible in a non-mucoid ancestor such as PAO1. Meanwhile several *P. aeruginosa* strains including isolates from CF lungs have been completely sequenced thus providing ample opportunities to perform functional genomics in sequenced *P. aeruginosa*. Strain PA14 [42] definitely is prime choice because it is representative for the most common clone in today *P. aeruginosa* population [37]. Standard doses, however, are lethal in rodent airway infection models which jeopardize comparative studies on acute and chronic lung infections (A. Bragonzi, personal communication). The TBCF10839 belonging to the major clone TB [37] may represent an adequate substitute. The mucoid isolate from a chronically infected CF lung with documented patient history is virulent in numerous invertebrate and mammalian infection models [34] and does not exhibit the common loss-of-function phenotypes that occur during the chronic adaptation to CF lungs [8]. At the time of isolation, the TB clone spread among patients with severe burn wounds, ventilated patients at ICUs and individuals with CF [38]. The vicinity of the evolutionary landscape of TBCF10839 should thus provide insight into the genomic capacity of a contagious, non-attenuated virulent *P. aeruginosa* that can colonize and persist in the disease habitats of contemporary infections in humans. The targets identified in this combined *in vitro* and *in vivo* screening already provide ample opportunity to analyze in further studies the complex interplay between dissociative behaviour, adaptive radiation, lifestyle and fitness of *P. aeruginosa*.

## Materials and Methods

### Strains, plasmids and growth conditions

Plasmids, strains and culture conditions are listed in Table 4. M9 minimal medium (10X: 0.48 M Na_2_HPO_4_, 0.22 M KH_2_PO_4,_ 85 mM NaCl, 0.18 M NH_4_Cl) contained 0.5% (v/v) glycerol as a carbon source for the screening of the STM transposon library. In general, *P. aeruginosa* or *E. coli* strains were routinely grown overnight (230 rpm) in Luria broth (LB) medium at 37°C. Five mL LB broth were inoculated with a toothpick of frozen bacterial stock solution and incubated for 24-48 h under the various conditions listed in Table 4. *E. coli* strains transfected with pME6010 [88] (DH5α) or pUCP20 constructs (XL10-Gold) were growing in the presence of 50 µg/mL tetracycline or 100 µg/mL ampicillin, respectively, and recombinant *P. aeruginosa* were cultured in the presence of 100 µg/mL tetracycline (pME6010) or 200 µg/mL carbenicillin (pUCP20).

**Table 4:** Strains, plasmids, oligonucleotide primers and culture conditions.

Strains	Genotype and/or source	Reference
*E. coli* DH5α	F^−^, φ80m80*1acZ*ΔM15, Δ(*lacYZA-arg*F), U169, *rec*A1, *end*A1, *hsd*R17, *(r_k−_; m_k+_), pho*A, *sup*E44, *λ^−^, thi*-1, *gyr*A96, *rel*A1	[97]
*E. coli* XL10-Gold	Δ(*mcr*A)183, Δ(*mcrCB-hsdSMR-mrr*)173, *end*A1, *sup*E44, *thi*-1, *rec*A1, *gyr*A96, *rel*A1, *lac*Hte, [F́, *pro*AB, *lacI^q^Z*Δ*M15*, Tn*10* (Tet^r^) Amy Cam^r^]	Stratagene
*P. aeruginosa* TBCF10839	CF airways, serotype 4; pyocin type: 1h, phage lysotype: F8, M4, PS2, PS24, PS31, 352, 46b/2, 1214, Col21, F7, F10, PS21, PS73, no plasmids. Hexadecimal SNP genotype [38]: 3C52.	[37]
**Plasmids**	**Genotype and/or source**	
pME6010	Shuttle vector for Gram-negative bacteria; Tc^r^	[88]
pUCP20	*Escherichia-Pseudomonas* shuttle vector; Ap^r^	[98]
pME6010::TB*pilY1*	pME6010 containing the BglI/EcoRI PCR product bearing the *pilY1* gene	This study
pME6010::TB*pilW*	pME6010 carrying the KpnI/EcoRI PCR product bearing the *pilW* gene	This study
pUCP20::TB*mvfR*	pUCP20 carrying the HindIII/SacI PCR product bearing the *mvfR* gene	This study
pUCP20::TB*pqsD*	pUCP20 carrying the HindIII/SacI PCR product bearing *pqsABCD* operon	This study
pUCP20::TB*edd*	pUCP20 carrying the HindIII/SacI PCR product bearing the *edd* gene	This study
pUCP20::TB*mqoB*	pUCP20 carrying the HindIII/SacI PCR product bearing the *mqoB* gene	This study
pUCP20::TBPA4131	pUCP20 carrying the HindIII/SacI PCR product bearing the PA4131 gene	This study
pUCP20::TBPA0785	pUCP20 carrying the HindIII/SacI PCR product bearing the PA0785-PA0787 genes	This study
**Primers**	**Sequence**	**Reference**
5′pilW_KpnI	GCCGGTACCCGACTTCTTCAAGGCCAAGG	This study
3′pilW_EcoRI	GCGAATTCCGCGCTGTTGTGCAGGGAAGT	
5′pilY1_Bgl	CGGAGATCTGGAACAACCTGCCCATTCCC	This study
3′pilY1_EcoRI	GCCGAATTCGAAGGTCTGGGGATCTTCGG	
5′mvfR_HindIII	GGATAAGCTTACACCTGAAGGCGCAACAGC	This study
3′mvfR_SacI	CTAGAGCTCCGGAAGGTTTCGACTGCCTG	
5′pqsD_HindIII	GGATAAGCTTGAAGCCTGCAAATGGCAGGC	This study
3′pqsD_SacI	CTAGAGCTCGACGCCAGGACCTGTACGTT	
5′edd_HindIII	GGATAAGCTTGCGTTCGAGACGATCCGATG	This study
3′edd_SacI	CTAGAGCTCCCGGCGCTTCTCTTGTTGTCG	
5′mqoB_HindIII	GGATAAGCTTCACTGAGCAACAGGCGATGCAGC	This study
3′mqoB_SacI	CTAGAGCTCCCTGTTTCGGTACCCTGGTGG	
5′PA4131_HindIII	GGATAAGCTTCAGGTAAAGGTACAGGCCGATG	This study
3′PA4131_SacI	CTAGAGCTCTCTCGTAGCGCTTCATCTTG	
5′PA0785_HindIII	GGATAAGCTTCAGTGGTGGAGACCGTCAGGTTG	This study
3′PA0785_SacI	CTAGAGCTCCTGCCAGTGCAGGTACTCAAG	
5′P1	GTACCCCACTAGTCCCAAGC	This study
5′P2	GTACCTCCACTCACCCAAGC	
5′ Y-linker	CTGCTCGAGCTCAAGCTTCG	[46]
5′ Tn*5*	TGCGTTCGGTCAAGGTTCTGG	This study
5′Y1	TTTCTGCTCGAGCTCAAGCTTCGAACGATGTACGGGGACACATG	[46]
5′Y2	TGTCCCCGTACATCGTTCGAACTACTCGTACCATCCACAT	
**Culture Conditions**	**Media (2% agar)**	
37°C	Luria broth (LB)	
	LB supplemented with 4 mM FeSO_4_	
	LB depleted from iron by Chelex-100 beads (Sigma) [96]	
	Minimal medium (M9)	
	Blood-agar (Columbia agar containing 5% sheep blood)	
	LB supplemented with Congo red (40 µg/mL)	
4°C, ambient temperature, 42°C	LB	

### Construction of the *P. aeruginosa* TBCF10839 STM transposon library

The experimental procedure is described in Text S1. Transposon mutagenesis in *P. aeruginosa* was performed with the plasposon pTnModOGm [43] carrying variable signature tags [34].

### DNA preparation

Genomic DNA from *P. aeruginosa* was isolated according to a protocol optimized for gram-negative bacteria [89]. The DNA was used as template for PCR, Y-linker method, plasmid rescue and Southern blotting.

### Sequencing of transposon flanking genes

Transposon–flanking sequences were amplified by the Y-linker method [46], [90]. Oligonucleotide sequences (Y-linker, Tn*5*MOD) are listed in Table 4. The Y-linker was prepared by the annealing of two oligonucleotides, Y1 and Y2. Genomic DNA of the mutants in aliquots of 1 µg was cut with 5 U NlaIII or SphI (New England Biolabs) for 3 h and ligated to the phosphorylated and annealed Y-linker with T4-DNA ligase at 25°C for 2 h. The resulting product was used as a template in a PCR with the Y- and pTnMOD-specific primers. The resulting PCR products were purified by agarose gel electrophoresis and extracted using a Qiagen Gel extraction Kit. Sequencing was done by QIAGEN using the pTnMOD-specific primer. The raw sequences were analysed by blastn search against the sequences of the predicted genes as well as the complete genome sequence of *P. aeruginosa* PAO1 [41] or PA14 [42].

For non-PAO1 genes, the plasposon was mobilized from the chromosome by plasmid rescue [43] to get longer stretches of transposon flanking sequence than accomplished by the Y-linker method. Ten µg genomic DNA of the transposon mutant was digested with 40 U PstI overnight at 37°C in 40 µl restriction buffer, purified by phenol/chloroform extraction, and the pellet was resuspended in 25 µl TE buffer. An aliquot of 500 ng PstI-digested genomic DNA was incubated with 1000 cohesive end ligation units of T4-DNA ligase for 6 h at 16°C in 25 µl ligase buffer (New England Biolabs). Fifty ng of ligated DNA were transformed into *E. coli* XL10-Gold and plasmid-harbouring cells were selected with gentamicin (30 µg/ml) on LB agar. The plasposon was purified from cultures of recombinant cells and sent for sequencing to QIAGEN.

### Complementation of *P. aeruginosa* mutants

Complementation *in trans* was performed by transformation of transposon mutants with recombinant plasmids pME6010 [88] for promoterless genes or pUCP20 for all other genes (Table 4). The genes with their own promoter regions, if applicable were amplified from TBCF10839 DNA by PCR with GoldStar polymerase (Eurogentec). Restriction-digested PCR products were ligated into the shuttle vector pME6010 or pUCP20. Correct size of the inserted sequence was verified by restriction digestion analysis. The plasmid was introduced into chemically competent *E. coli* (DH5α or XL10-Gold) and subsequently into the respective *P. aeruginosa* transposon mutant by electroporation [91].

### Murine infection experiments

Prior to animal experiments, bacteria were grown in LB broth overnight at 37°C (230 rpm) to stationary phase. The bacteria were pelleted by centrifugation (5000×g, 10 min), washed twice with sterile phosphate buffered saline (PBS) and the optical density of the bacterial suspension was adjusted by spectrophotometry at 578 nm. The intended number of cfu was extrapolated from a standard growth curve, and appropriate dilutions with sterile PBS were made to prepare the inoculum for the mice. To verify the correct dilution, an aliquot was serially diluted on LB agar plates. Ten to 12 week old female mice of the inbred strain C3H/HeN (Charles River, Sulzfeld, Germany) were inoculated with 30 µl of this bacterial suspension containing 7.5×10^6^ cfu of the different *P. aeruginosa* mutants via view controlled intratracheal instillation. This noninvasive application technique via catheter [35] allows controlled delivery of the bacteria to the lungs. During the experiments mice were maintained in microisolator cages with filter top lids at 21±2°C, 50±5% humidity and 12 h light-dark-cycle. They were supplied with autoclaved, acidulated water and fed ad libitum with autoclaved standard diet. Mice were sacrificed by 48 h for the evaluation of lung histology or the determination of cfu in homogenized organs (lungs, liver, spleen and brain).

Prior to the start of the experiments animals were acclimatized for at least seven days. All animal procedures were approved by the local District Governments and carried out according to the guidelines of the German law for the protection of animal life.

### Screening of pools of STM mutants for survival *in vivo*

Transposon mutants with different signature tags were separately grown in LB (37°C) overnight and pooled directly before mice infection. This pool was split into an aliquot of 100 µl of bacterial suspension that was cultivated on LB agar or LB broth for 48 h at 37°C (control) and another aliquot of 30 µl (7.5×10^6^ cfu) that was used for the intratracheal mice infection (experiment). After 48 h of infection, mice were sacrificed and organs (lung, liver, spleen) were homogenised. Bacteria from the homogenised organs were recovered on LB and LB agar at 37°C overnight. In parallel, bacteria from the control plates were collected and incubated on LB and LB agar at 37°C overnight in the same incubator. Genomic DNA was prepared from both control and experiment and a PCR was performed to amplify the signature tags (primers P1 and P2 (Table 4; 35 cycles; 20 s, 58°C; 20 s, 72°C; 30 s, 94°C; 10 s ramp between each step). The PCR products were digested for 16 h with HindIII and the specific 40 bp sequence tags were separated from the common flanking 20 bp sequences by polyacrylamide gel electrophoresis (10% gel (19+1 acrylamide/bis-acrylamide) in TBE buffer). The 40 bp sequence tags were cut out from the gel and purified (QIAGEN). The 40 bp sequences were labelled with DIG-ddUTP using a terminal transferase (Roche) and hybridized onto dot blots. The dot blots were prepared as follows: 80 µl of the PCR products amplified from each single mutant were mixed with 40 µl of 3M NaOH and 280 µl of TE buffer and denatured at 65°C for 30 min. After transfer to ice, 400 µl of 2M CH_3_COONH_4_ were added and after short incubation, an aliquot of 95 µl of the DNA solution were applied to a Minifold-DOT-vacuum-blot device (Schleicher & Schuell) to immobilize the DNA on a Hybond N^+^ membrane. Dried and cross-linked membranes were pre-hybridized with 10 ml hybridization buffer (0.5 M NaH_2_PO_4_×2H_2_O, 7% SDS, 1mM EDTA, 0.5% blocking reagent (Roche), pH 7.2) for 2 h at 58°C and afterwards hybridized with 40 ml of hybridization buffer containing denatured DIG-labelled probes for 16–24 hrs at 58°C in a hybridization oven. Non-specifically bound probe solution was removed by several washing steps and hybridization signals were detected by washing the membrane with anti-DIG–alkaline phosphatase (Roche) and CDP Star [92]. Chemoluminescence signals were detected on X-ray films and quantified by PCBAS, version 2.09f (raytest Isotopenmeßgeräte GmbH). Signal intensity of each dot was compared with that of the corresponding signal of the probe prepared from pooled bacteria grown in parallel on LB agar without *in vivo* selection. Hybridization signals out of the 95% confidence interval of the mean were interpreted to be significantly different from the average signal. These mutants were retested and transposon mutants with consistently strong differences in their ability to survive were selected for further examinations.

### Phenotype MicroArrays™

*P. aeruginosa* TBCF10839, PAO1 and selected mutants were analyzed in their metabolic profile on Phenotype MicroArray plates (PM1-PM5) for carbon, phosphorus, nitrogen and sulphur catabolism (BiOLOG Inc., Hayward, Ca.). The method monitors the utilization of various C, P, N and S sources by the kinetics of the reduction of a tetrazolium dye [93], [94]. To verify the BiOLOG data [93], the mutants were re-tested in-house by growth experiments with selected nutrients in 96-well plates. The reference minimal medium contained 100 mM NaCl, 30 mM triethanolamine HCI (pH 7.1), 25 mM sodium pyruvate, 5.0 mM NH_4_Cl, 2.0 mM NaH_2_PO_4_, 0.25 mM Na_2_SO4, 0.05 mM MgCl_2_, 1.0 mM KCl, 1.0 mM ferric chloride, and 0.01% tetrazolium violet. To test the utilization of specific compounds as C, P, N or S source, carbon-free, nitrogen-free, phosphorus-free, or sulfur-free versions of the reference minimal medium were used. Supplementation was performed with 25–100 µM sulfur source, 0.1–1 mM phosphorus source, 2–20 mM nitrogen source or 5–50 mM carbon source, respectively (Figure 7, Figure S1).

Strains were grown overnight at 37°C on minimal medium. Cells were picked from the agar surface with a sterile cotton swab and suspended in 10–15 ml of appropriate minimal medium. The cell density was adjusted to OD_578nm_ = 0.02 and cells were directly inoculated into 96 well plates containing 100 µl of minimal medium. All microplates were incubated at 37°C and monitored for color change at 490 nm with an Elisa reader. Readings were recorded after 12, 18 and 24 hours. All tests were performed in triplicate.

### HAQ detection and quantification [49]

*P. aeruginosa* strains were grown in a 50 mL-flask in 10 ml Brain Heart-Infusion medium with constant shaking at 37°C for 18 h. Five ml of the culture were extracted with 5 mL dichlormethane by vigorous shaking and the liquid phases were separated by centrifugation at 5000×g for 10 min. Two mL of the organic phase containing HAQs and pyocyanin as major components were dried by evaporation. The pellet was resuspended in 50 µL methanol. Two to four µL thereof were separated on Silica Gel 60 F254 TLC plates with 5% methanol/95% dichlormethane as the mobile phase. Fluorescent spots were visualized under UV light and photographed. Starting from 4-hydroxy-2-heptylquinoline, PQS was synthesized by the procedures described by Pesci et al. [95] and used as standard. Molecular identity of PQS was verified by trimethylsilylation [50/50 pyridine+ (bis-N,O-trimethylsilyl trifluoroacetamide+1% trimethylchlorosilane), 70°C, 1 h] and subsequent analysis on a Thermo-Finnigan GCQ ion trap mass spectrometer (Finnigan MAT, San Jose, CA) running in the positive ion EI mode equipped with a 30 m DB5 capillary column.

## Supporting Information

Text S1 Construction of the Signature Tagged Mutagenesis (STM) transposon library in Pseudomonas aeruginosa TBCF10839.
(0.04 MB DOC)

Table S1 STM Tn5 TBCF10839 colony morphology variants: media and culture conditions used in the study that led to detectable changes in morphotype.
(0.12 MB DOC)

Table S2 Oligonucleotide dot blot templates to recognize P. aeruginosa STM transposon mutants in murine airway infection competition experiments
(0.11 MB DOC)

Table S3 Phenotype MicroArrays (PMs) of P. aeruginosa TBCF10839 Tn5::motC.
(0.03 MB DOC)

Table S4 Phenotype MicroArrays (PMs) of P. aeruginosa TBCF10839 Tn5::mqoB.
(0.03 MB DOC)

Table S5 Phenotype MicroArrays (PMs) of P. aeruginosa TBCF10839 Tn5::edd.
(0.05 MB DOC)

Figure S1 Metabolic phenotyping. Growth of P. aeruginosa strains TBCF10839 (open bar), PAO1 (grey bar) and TBCF10839 Tn5::TBmotC (A, dashed bar), TBCF10839 Tn5::TbmqoB (B, dashed bar), TBCF10839 Tn5::TBedd (C, dashed bar) at 37oC in standard minimal mineral medium supplemented with nutrients as carbon, nitrogen, phosphor or sulphur source other than shown in Figure 7 of the main manuscript. The ordinate indicates the optical density at 490 nm. The number below the triple bars indicates the time of culturing of 12 h (left), 18 h (middle) and 24 h (right). The initial bacterial inoculum had an OD578 of 0.02 in 100 µL minimal medium supplemented with the indicated source. All growth experiments were performed in triplicate in 96-well plates.
(0.48 MB TIF)
