# Supplementary material for: Fitness of Isogenic Colony Morphology Variants of Pseudomonas aeruginosa in Murine Airway Infection
Source: PLoS One. 2008 Feb 27;3(2):e1685. doi: 10.1371/journal.pone.0001685 (PMC2246019; doi:10.1371/journal.pone.0001685)
Supplement: Text S1 — Construction of the Signature Tagged Mutagenesis (STM) transposon library in Pseudomonas aeruginosa TBCF10839. (0.04 MB DOC) [file pone.0001685.s001.doc]

#### Supporting Text S1

#### Construction of the Signature Tagged Mutagenesis (STM) Transposon Library in *Pseudomonas aeruginosa* TBCF10839.

**1. Strains and Plasmids**

Plasmids, *P. aeruginosa*- and *E. coli*- strains are listed in Table 1. Both bacterial species were grown in Luria broth (LB) medium at 37°C. *E. coli* HB101 with pRK2013 were grown in LB with 25 µg/ml kanamycin, for *E. coli* DH5α with pTnModOGm [1] (and its derivative pTnModOGm SigTag) 15 µg/ml gentamicin were added to the growth media. To select for stable transposon mutants of *P. aeruginosa*, the bacteria were grown on M9 minimal medium with 25 µg/ml gentamicin and 0.5 % glycerol as sole carbon source.

**2. DNA Preparation**

Plasmid DNA from transformed *E. coli* was prepared according to a modified alkaline lysis [2] or with Qiagen kits. Genomic DNA from *P. aeruginosa* was isolated according to a protocol optimized for gram- negative bacteria [3]. The DNA was used as a template for PCR reactions or for Southern Blots.

**3. Generation of Donor- Plasmids for STM in *P. aeruginosa***

The following signal sequence was synthesized:

CTGGGTACCCCACTAGTCCCAAGCTTV_40_AAGCTTGGGTGAGTGGAGGTACCGTG

*Kpn*I *Spe*I *Hin*dIII *Hin*dIII *Kpn*I

(V = A, G, C)

Primers:

P0: 5’-CACGGTACCTCCACTCACCC

P1: 5’-GTACCCCACTAGTCCCAAGC; P2: 5’-GTACCTCCACTCACCCAAGC

The artificial single strand DNA was complemented with primer P0 (annealing 20 s, 61°C; elongation 20 s, 72°C), digested with *Kpn*I, ligated into pTnMod-OGm [1] resulting in pTnMod-OGm -SigTag and transformed into *E. coli* DH5α [4]. (Because of the high multiplicity of the restriction sites were more than 50 U/µg signal sequence needed for a complete digestion of the DNA.) 192 *E. coli* strains with different signal sequences were arrayed three-dimensionally (4 x 6 x 8). To exclude pTnMod-OGm -Signature Tags with non- discriminatory signal intensities under the chosen hybridization conditions, signature tags pooled from the planes, rows and columns of the array, respectively, were amplified by PCR, digested with *Hin*dIII (with the above mentioned parameters) and purified by gel filtration. Probes were prepared by using a DIG- ddUTP 3´-end labelling kit (Roche), hybridized against dot blots containing all 192 pTnMod-OGm SigTags (62°C, 16 h) and detected with CDP- Star (Tropix, Roche). 48 plasmids with comparable signal strength and specific hybridization signals were chosen for the construction of the STM- library in *P. aeruginosa.*

**4. Generation of *P. aeruginosa*  TBCF10839- Transposon Mutants**

*P.aeruginosa* TBCF10839 were incubated on Columbia agar containing 5% sheep blood at 42°C for five days and transferred to new agar plates each day. After this preincubation period, the *P. aeruginosa* were resuspended in 10 mM MgSO_4_ to an optical density of 1 OD (λ= 578 nm). Overnight cultures of *E. coli* carrying the pTnMod-OGm -SigTag (donor- strain) and pRK2013 (helper- strain) [5] were also resuspended in 10 mM MgSO_4_ to a final concentration of 1 OD. *P. aeruginosa*, donor- and helper- strains were mixed in a ratio of 1 : 10 : 10, plated on LB agar and were incubated overnight at 37°C. The bacteria were recovered from the plate and resuspended in 5 ml 10 mM MgSO_4_ and *P. aeruginosa* transposon mutants were selected on M9 -minimal media containing 0.2 % glycerol as a sole carbon source and 25 µg/ml gentamicin. The plates were maintained first for 3 days at 37°C and then for a further 6 weeks at 4°C. This procedure ensured complete suppression of contaminating viable *E. coli*, because *E. coli* can ferment glycerol but does not grow on glycerol in M9 medium and loses viability during extended storage at 4 °C.

**Comments**

a. Signature Tags. We used V_40_ (V=A,G,C) as the signature tag, because first, it matches the GC- content of the bulk *P. aeruginosa* genome and second, V_40_ sequences contain virtually no recognition sites for the common restriction endonucleases.

b. We constructed the library with the plasposon pTnModOGm [1] because it is episomally stable in most *E. coli* and the transposed sequence can be mobilized from the *P. aeruginosa* host by plasmid rescue.

c. Selection of tags. Of 192 signature tagged plasmids, about 10% of the synthesized oligonucleotide tags cross- hybridized with others and another 50% of the tags yielded low signals under stringent hybridization conditions. The library was constructed with the remaining 40% of tags.

**References**

1. Dennis JJ, Zylstra GJ (1998) Plasposons: modular self-cloning minitransposon derivatives for rapid genetic analysis of gram-negative bacterial genomes. Appl Environ Microbiol 64: 2710-2715.

2. Birnboim HC, Doly J (1979) A rapid alkaline extraction procedure for screening recombinant plasmid DNA. Nucleic Acids Res 7: 1513-1523.

3. Chen WP, Kuo TT (1993) A simple and rapid method for the preparation of gram negative bacterial genomic DNA. Nucleic Acids Res 21: 2260.

4. Inoue H, Nojima H, Okayama H (1990) High efficiency transformation of *Escherichia coli* with plasmids*.* Gene 96: 23-28.

5. Figurski D, Helinski D (1979) Replication of an origin-containing derivative of plasmid RK2 dependent on a plasmid function provided in trans. Proc Natl Acad Sci U S A 76: 1648-1652.

**Table 1: Bacterial strains and plasmids**

**Strains Genotype and/or Source Reference**

*E.coli* DH5α F’ (*endA1 hsdR17*(r_K_-m_K_+)sup*E44 thi-1 recA1*

*gyrA relA1* Δ*(lacYZA-argF)_U169_(m80lacZΔM15)*

*E.coli* HB101 *leuB6 thi-1 lacY1 hsdS_B_20 recA , rpsL20 ara-14*   *galK2 xyl-5 mtl-1 supE44 mcrB_B_* Δ(*gpt-proA*)*62*

*P. aeruginosa* TBCF10839: CF airways, serotype 4; pyocin type: 1h, phage lysotype:F8, M4, PS2, PS24, PS31, 352,

46b/2, 1214, Col21, F7, F10, PS21, PS73,

no plasmids

**Plasmids**

pTnModOGm *Gm*^R^, mini-Tn*5* plasposon [1]

pTnModOGm SigTag pTnModOGm with Signature Tag This work

pRK2013 *Km*^R^, conjugation vector for triparental mating [5]
