# Supplementary material for: Fitness of Isogenic Colony Morphology Variants of Pseudomonas aeruginosa in Murine Airway Infection
Source: PLoS One. 2008 Feb 27;3(2):e1685. doi: 10.1371/journal.pone.0001685 (PMC2246019; doi:10.1371/journal.pone.0001685)
Supplement: Table S1 — STM Tn5 TBCF10839 colony morphology variants: media and culture conditions used in the study that led to detectable changes in morphotype. (0.12 MB DOC) [file pone.0001685.s002.doc]

**Table S1.** STM Tn*5* TBCF10839 colony morphology variants: media and culture conditions^1^ used in the study that led to detectable changes in morphotype.

| **PAO1 gene number of transposon insertion** | **1**  **LB agar** | **2**  **LB agar**  **+**  **Congo red dye** | **3**  **LB agar supplemented with iron**  **(Fe^2+^)** | **4**  **LB agar depleted from iron** | **5**  **LB agar**  **/**  ***LB agar**  **+**  **Congo red dye** | **6**  **LB agar** | **7**  **LB agar^1^** | **8**  **Minimal medium**  **(M9 agar)** | **9**  **Blood-agar** |
| --- | --- | --- | --- | --- | --- | --- | --- | --- | --- |
|  | **37^o^C** | | | | **22 – 25^o^C** | **42^o^C** | **4^o^C** | **37^o^C** | |
| PA0424 | **#** | **#** | **#** | **#** | **#** | **#** |  | **#** | **#** |
| PA2028 | **#** | **#** | **#** | **#** | **#** | **#** |  | **#** | **#** |
| PA2122 | **#** | **#** | **#** | **#** | **#** | **#** |  | **#** | **#** |
| PA3462 | **#** | **#** | **#** | **#** | **#** | **#** |  | **#** | **#** |
| PA3748 | **#** | **#** | **#** | **#** | **#** | **#** |  | **#** | **#** |
| PA4190 | **#** | **#** |  | **#** | **#** | **#** |  |  | **#** |
| PA4489 | **#** | **#** | **#** | **#** | **#** | **#** |  | **#** | **#** |
| PA5524 | **#** | **#** | **#** | **#** | **#** | **#** |  | **#** | **#** |
| A1 | **#** | **#** | **#** | **#** | **#** | **#** |  | **#** | **#** |
| A2 | **#** | **#** | **#** | **#** | **#** | **#** |  | **#** | **#** |
| A3 | **#** | **#** | **#** | **#** | **#** | **#** |  | **#** | **#** |
| PA0999 |  | **#** | **#** |  |  |  |  |  | **#** |
| PA1003 |  | **#** | **#** |  |  |  |  |  | **#** |
| PA2361 |  | # | # |  |  |  |  |  | # |
| PA4915 |  | **#** | **#** |  |  |  |  |  | **#** |
| PA2537 |  | **#** |  |  |  |  |  |  |  |
| PA2579 |  | # |  |  |  |  |  |  |  |
| PA2838 |  | **#** |  |  |  |  |  |  |  |
| PA4734 |  | **#** |  |  |  |  |  |  |  |
| PA4552 | **#** | **#** |  |  |  |  |  |  |  |
| PA4554 | **#** | **#** |  |  |  |  |  |  |  |
| PA0413 |  |  |  |  | **# / #*** |  |  |  |  |
| PA0415 |  |  |  |  | **# / #*** |  |  |  |  |
| PA1846 |  |  |  |  | **# / #*** |  |  |  |  |
| PA4954 |  |  |  |  | **# / #*** |  |  |  |  |
| PA2388 | **#** | **#** |  |  |  |  |  |  |  |
| PA2391 | **#** | **#** |  |  |  |  |  |  |  |
| PA3194 | **#** | **#** |  |  |  | **#** |  |  | **#** |
| PA4640 | **#** | **#** |  |  |  | **#** |  |  | **#** |
| H1 | **#** | **#** | **#** |  |  |  |  |  | **#** |
| PA0482 |  | x |  |  |  |  |  |  |  |
| PA0728 |  | x |  |  |  |  |  |  |  |
| PA0785 |  | x |  |  |  |  |  |  |  |
| PA0920 |  | x |  |  |  |  |  |  | x |
| PA1589 |  | x |  |  |  |  |  |  |  |
| PA1633 |  | x |  |  |  |  |  |  |  |
| PA1823 |  | x |  |  |  |  |  |  | x |
| PA2706 |  | x |  |  |  |  |  |  |  |
| PA2946 | x | x |  |  |  |  |  |  |  |
| PA3012 |  | x |  |  |  |  |  |  |  |
| PA3238 |  | x |  |  |  |  |  |  |  |
| PA3239 |  | x |  |  |  |  |  |  |  |
| PA3804 |  | x |  |  |  |  |  |  | x |
| PA4131 |  | x |  | x |  |  |  |  |  |
| PA4703 |  | x |  |  |  |  |  |  |  |
| PA4797 |  | x |  |  |  |  |  |  |  |
| PA4949 |  | x |  |  |  |  |  |  |  |
| PA4951 | x | x |  |  |  |  |  |  |  |
| PA5121 |  | x |  |  |  |  |  | x |  |
| PA5231 |  | x |  |  |  |  |  |  |  |
| PA5546 | x | x |  |  |  |  |  |  |  |
| PA5563 |  | x |  |  |  |  |  |  |  |
| UK1 |  | x |  |  |  |  |  |  |  |
| PR1 |  | x |  |  |  |  |  |  |  |
| PR2 |  | x |  |  |  |  |  |  | x |

# Stable morphotype distinct from wild type

X Unstable morphotype with rapid reversion to wild type morphotype

**^1^** Cultures of conditions 1–6, 8, 9 were examined after 48 h, cultures in LB agar at 4^o^C were examined every day for a period of 21 days.

**Genes having no homolog in PAO1 genome:**

A1 – *topA,* topoisomerase IA, *P. aeruginosa* 2192

A2 – *fpvAIII,* siderophore receptor for type III ferripyoverdine, *P. aeruginosa* strain 59.20

A3 - *phiCTXp40,* *Pseudomonas* phage phiCTX, hypothetical protein ORF37

H1 – Unknown function gene (non-coding sequence) of the genomic “island” of *P. aeruginosa* 2192; gene is located in the range 3291786 – 3291972 of *P. aeruginosa* 2192 genome.

UK1 – Unknown gene (no homologs in PAO1 and other genomes)

**Promoter regions:**

PR1 - Promoter region of PA1266 gene

PR2 - Promoter region of PA3782 gene
