# Supplementary material for: Fitness of Isogenic Colony Morphology Variants of Pseudomonas aeruginosa in Murine Airway Infection
Source: PLoS One. 2008 Feb 27;3(2):e1685. doi: 10.1371/journal.pone.0001685 (PMC2246019; doi:10.1371/journal.pone.0001685)
Supplement: Table S2 — Oligonucleotide dot blot templates to recognize P. aeruginosa STM transposon mutants in murine airway infection competition experiments (0.11 MB DOC) [file pone.0001685.s003.doc]

**Table S2.** Oligonucleotide dot blot templates to recognize *P. aeruginosa* STM transposon

mutants in murine airway infection competition experiments.^+^

**Experiment 2**

**C**

**B**

**A**

PA5121

PA4131

PA1633

PA5563

PA5546

PA2706

PA2579

PA4797

PA5231

PA3194

PA2838

A3

UK1

PA4954

PA2388

PA2588

PA4734

PA4640

PA0413

PA0999

PA1003

PA2361

PA4915

PA4554

PA4552

**12**

**11**

**10**

**9**

**8**

**7**

**6**

**5**

**4**

**3**

**2**

**1**

**B**

**A**

PA4552

PA4554

PA5563

PA2361

PA0999

PA4954

PA3239

PA1846

PA4951

PA3012

PA4103

PA4190

PA2706

PA3194

A3

PA4131

**12**

**11**

**10**

**9**

**8**

**7**

**6**

**5**

**4**

**3**

**2**

**1**

**Experiment 3**

**B**

**A**

PA2028

PA1633

PA4554

A1

PA2838

PA1823

PA5546

PA4640

PA0785

PA2122

PA1003

PA0424

PA0415

PA2388

PA4734

PA2391

**12**

**11**

**10**

**9**

**8**

**7**

**6**

**5**

**4**

**3**

**2**

**1**

**Experiment 4**

PA3239

PA2122

PA3748

**B**

PA3462

PA4949

A2

PA3238

PA2537

PA4951

PA0482

PA4489

PA5231

PA1589

PA5524

PA4916

**A**

**12**

**11**

**10**

**9**

**8**

**7**

**6**

**5**

**4**

**3**

**2**

**1**

**Experiment 5**

**Experiment 6**

**A**

A3

PA4640

PA5563

PA2537

PA2838

PA0999

PA3194

PA5231

PA2388

PA2588

**10**

**9**

**8**

**7**

**6**

**5**

**4**

**3**

**2**

**1**

**A**

PA4554

PA4552

PA4916

PA5546

PA4734

PA1589

PA4954

PA1846

PA3194

PA4131

**10**

**9**

**8**

**7**

**6**

**5**

**4**

**3**

**2**

**1**

**Experiment 1***

^+^ The PA number of the transposon-inactivated gene that carries the signature tag complementary to the oligonucleotide on the dot blot is shown for each infection experiment (see Figure 4 for the primary dot blot hybridization data).

* The same row letters (A - C) and column numbers (1 -12) were assigned to identical mutants

recovered from control, lung, spleen and liver.
