# Supplementary material for: Fitness of Isogenic Colony Morphology Variants of Pseudomonas aeruginosa in Murine Airway Infection
Source: PLoS One. 2008 Feb 27;3(2):e1685. doi: 10.1371/journal.pone.0001685 (PMC2246019; doi:10.1371/journal.pone.0001685)
Supplement: Table S3 — Phenotype MicroArrays (PMs) of P. aeruginosa TBCF10839 Tn5::motC. (0.03 MB DOC) [file pone.0001685.s004.doc]

***Table S3.*** *Phenotype MicroArrays (PMs) of* *P. aeruginosa TBCF10839 Tn*5::motC.

| **Test*^a^*** | **Difference*^b^*** | | **Mode** **of action** |
| --- | --- | --- | --- |
|  | **TBCF10839** | **PAO1** |  |
| p-Hydroxy-Phenylacetic Acid | 83 |  | C-source |
| L-Malic Acid | 68 |  | C-source |
| a-D-Glucose |  | 85 | C-source |
| p-Hydroxy-Phenylacetic Acid |  | 76 | C-source |
| L-Glutamine | 104 |  | N-source |
| Acetamide | 98 |  | N-source |
| L-Phenylalanine | 66 |  | N-source |
| Histamine | 51 |  | N-source |
| Ammonia | 50 |  | N-source |

(***a***) Chemicals were tested in 96-well PMs. (***b***) The OmniLog-PM software generates time course curves for respiration (tetrazolium color formation) and calculates differences between the areas for mutant and control cells. The units are arbitrary. Positive values indicate that the mutant showed greater rates of respiration than the wild type strains (TBCF10839 and PAO1). The differences are averages of pairwise comparisons. All assays were performed in duplicate.
