# Supplementary material for: Fitness of Isogenic Colony Morphology Variants of Pseudomonas aeruginosa in Murine Airway Infection
Source: PLoS One. 2008 Feb 27;3(2):e1685. doi: 10.1371/journal.pone.0001685 (PMC2246019; doi:10.1371/journal.pone.0001685)
Supplement: Table S4 — Phenotype MicroArrays (PMs) of P. aeruginosa TBCF10839 Tn5::mqoB. (0.03 MB DOC) [file pone.0001685.s005.doc]

***Table S4.*** *Phenotype MicroArrays (PMs) of* *P. aeruginosa TBCF10839 Tn*5::mqoB.

| **Test*^a^*** | **Difference*^b^*** | | **Mode of action** |
| --- | --- | --- | --- |
|  | **TBCF10839** | **PAO1** |  |
| L-Malic Acid |  | -111 | C-source |
| Acetic Acid | -76 | -74 | C-source |
| Malonic Acid | -54 |  | C-source |
| Fumaric Acid | -52 |  | C-source |
| L-Asparagine | -66 |  | N-source |
| Adenosine | -66 |  | nutrient stimulation |
| Positive Control* | -61 |  | nutrient stimulation |
| Tween 80 | -57 |  | nutrient stimulation |
| Inosine + Thiamine | -50 |  | nutrient stimulation |
| 2-Hydroxyethane Sulfonic Acid | -70 |  | S-source |
| L-Methionine Sulfoxide | -62 |  | S-source |
| Methane Sulfonic Acid | -58 |  | S-source |

(***a***) Chemicals were tested in 96-well PMs. (***b***) The OmniLog-PM software generates time course curves for respiration (tetrazolium color formation) and calculates differences between the areas for mutant and control cells. The units are arbitrary. Negative values indicate that the control showed greater rates of comparisons respiration than the mutant. The differences are averages of pairwise comparisons. All assays were performed in duplicate.

*****The positive control contained LB broth without any supplement.
