# Supplementary material for: Fitness of Isogenic Colony Morphology Variants of Pseudomonas aeruginosa in Murine Airway Infection
Source: PLoS One. 2008 Feb 27;3(2):e1685. doi: 10.1371/journal.pone.0001685 (PMC2246019; doi:10.1371/journal.pone.0001685)
Supplement: Table S5 — Phenotype MicroArrays (PMs) of P. aeruginosa TBCF10839 Tn5::edd. (0.05 MB DOC) [file pone.0001685.s006.doc]

***Table S5.*** *Phenotype MicroArrays (PMs) of* *P. aeruginosa TBCF10839 Tn*5::edd.

| **Test*^a^*** | **Difference*^b^*** | | **Mode** **of action** |
| --- | --- | --- | --- |
|  | **TBCF10839** | **PAO1** |  |
| L-Malic Acid |  | -108 | C-source |
| D-Gluconic Acid | -106 |  | C-source |
| D-Glucose | -76 |  | C-source |
| L-Aspartic Acid | -68 |  | C-source |
| Xanthine | -55 | -76 | N-source |
| Adenosine | -57 | -73 | N-source |
| Uric Acid |  | -65 | N-source |
| Xanthosine |  | -61 | N-source |
| Acetamide | -111 |  | N-source |
| Guanosine | -71 |  | N-source |
| N-Acetyl-D-Glucosamine | -68 |  | N-source |
| Cytidine | -61 |  | N-source |
| D-Alanine | -55 |  | N-source |
| L-Asparagine | -51 |  | N-source |
| D-(+)-Glucose |  | -120 | nutrient stimulation |
| N-Acetyl D-Glucosamine |  | -107 | nutrient stimulation |
| Deferoxamine Mesylate |  | -103 | nutrient stimulation |
| Riboflavin |  | -101 | nutrient stimulation |
| Thiamine |  | -88 | nutrient stimulation |
| Putrescine | -82 |  | nutrient stimulation |
| Positive Control* | -65 |  | nutrient stimulation |
| 2`-Deoxy-Adenosine | -56 |  | nutrient stimulation |
| L-Asparagine | -55 |  | nutrient stimulation |
| Tween 80 | -54 |  | nutrient stimulation |
| Phosphoryl Choline |  | -143 | P-source |
| D-Glucose-6-Phosphate |  | -70 | P-source |
| D-Glucose-1-Phosphate |  | -68 | P-source |
| Pyrophosphate | -64 |  | P-source |

(***a***) Chemicals were tested in 96-well PMs. (***b***) The OmniLog-PM software generates time course curves for respiration (tetrazolium color formation) and calculates differences between the areas for mutant and control cells. The units are arbitrary. Negative values indicate that the control showed greater rates of comparisons respiration than the mutant. The differences are averages of pairwise comparisons. All assays were performed in duplicate.

*****The positive control contained LB broth without any supplement.
