# Supplementary figures and images for: Fitness of Isogenic Colony Morphology Variants of Pseudomonas aeruginosa in Murine Airway Infection
Source: PLoS One. 2008 Feb 27;3(2):e1685. doi: 10.1371/journal.pone.0001685 (PMC2246019; doi:10.1371/journal.pone.0001685)

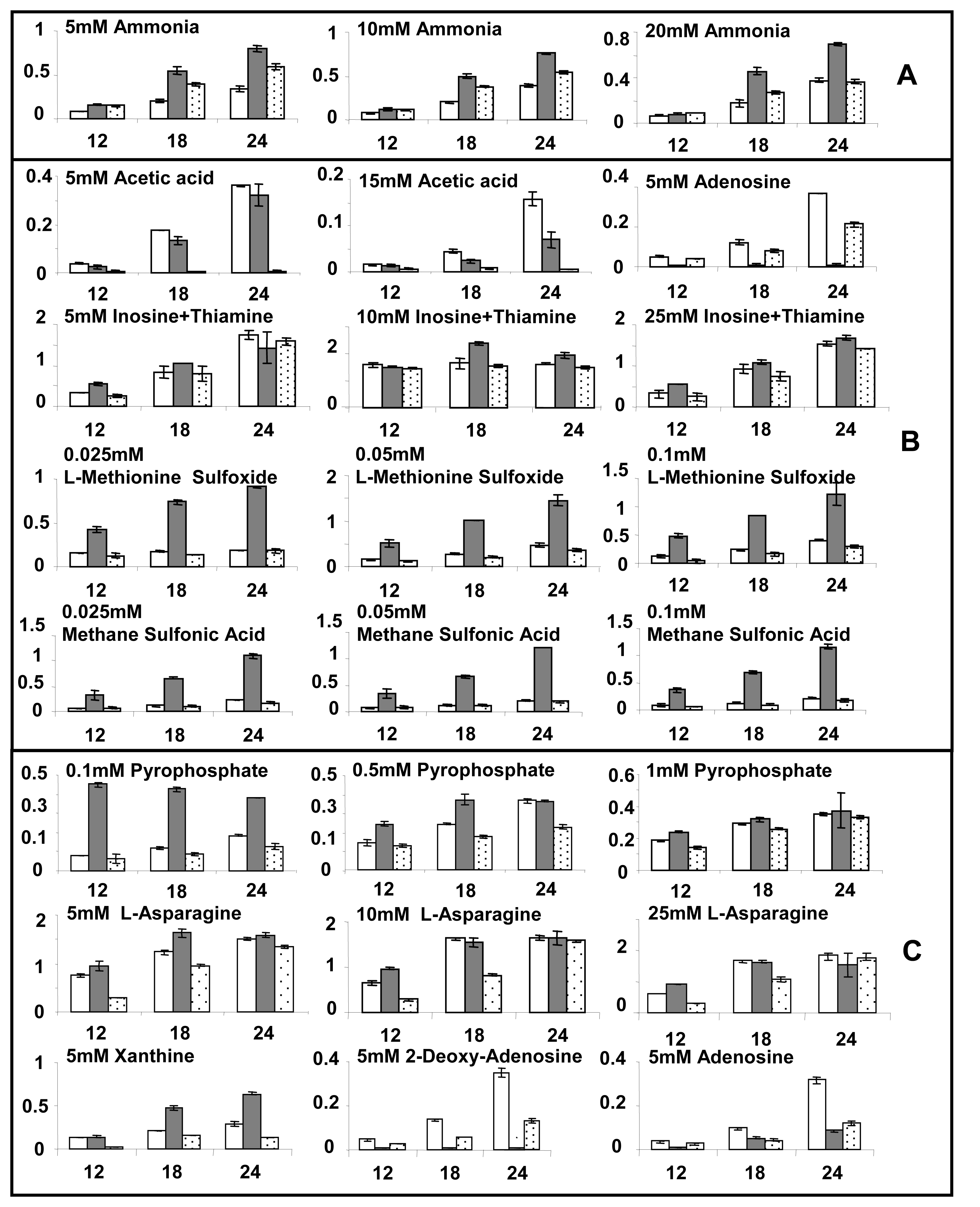

Supplement: Figure S1 — Metabolic phenotyping. Growth of P. aeruginosa strains TBCF10839 (open bar), PAO1 (grey bar) and TBCF10839 Tn5::TBmotC (A, dashed bar), TBCF10839 Tn5::TbmqoB (B, dashed bar), TBCF10839 Tn5::TBedd (C, dashed bar) at 37oC in standard minimal mineral medium supplemented with nutrients as carbon, nitrogen, phosphor or sulphur source other than shown in Figure 7 of the main manuscript. The ordinate indicates the optical density at 490 nm. The number below the triple bars indicates the time of culturing of 12 h (left), 18 h (middle) and 24 h (right). The initial bacterial inoculum had an OD578 of 0.02 in 100 µL minimal medium supplemented with the indicated source. All growth experiments were performed in triplicate in 96-well plates. (0.48 MB TIF) [file pone.0001685.s007.tif]
